# Genome-Guided Analysis of *Clostridium ultunense* and Comparative Genomics Reveal Different Strategies for Acetate Oxidation and Energy Conservation in Syntrophic Acetate-Oxidising Bacteria

**DOI:** 10.3390/genes9040225

**Published:** 2018-04-23

**Authors:** Shahid Manzoor, Anna Schnürer, Erik Bongcam-Rudloff, Bettina Müller

**Affiliations:** 1 Department of Information Technology, University of the Punjab, Lahore 54 590, Pakistan; shahid.dit.grw@pu.edu.pk; 2 BioCenter, Department of Molecular Sciences, Box 7015, Swedish University of Agricultural Sciences, SE 750 07 Uppsala, Sweden; Anna.Schnurer@slu.se; 3 SLU-Global Bioinformatics Centre, Department of Animal Breeding and Genetics Science, Swedish University of Agricultural Sciences, SE 750 07 Uppsala, Sweden; Erik.Bongcam@slu.se

**Keywords:** syntrophic acetate oxidation, hydrogen production, biogas process, energy conservation

## Abstract

Syntrophic acetate oxidation operates close to the thermodynamic equilibrium and very little is known about the participating organisms and their metabolism. *Clostridium ultunense* is one of the most abundant syntrophic acetate-oxidising bacteria (SAOB) that are found in engineered biogas processes operating with high ammonia concentrations. It has been proven to oxidise acetate in cooperation with hydrogenotrophic methanogens. There is evidence that the Wood-Ljungdahl (WL) pathway plays an important role in acetate oxidation. In this study, we analysed the physiological and metabolic capacities of *C. ultunense* strain Esp and strain BS^T^ on genome scale and conducted a comparative study of all the known characterised SAOB, namely *Syntrophaceticus schinkii*, *Thermacetogenium phaeum*, *Tepidanaerobacter acetatoxydans*, and *Pseudothermotoga lettingae*. The results clearly indicated physiological robustness to be beneficial for anaerobic digestion environments and revealed unexpected metabolic diversity with respect to acetate oxidation and energy conservation systems. Unlike *S. schinkii* and *Th. phaeum*, *C. ultunense* clearly does not employ the oxidative WL pathway for acetate oxidation, as its genome (and that of *P. lettingae*) lack important key genes. In both of those species, a proton motive force is likely formed by chemical protons involving putative electron-bifurcating [Fe-Fe] hydrogenases rather than proton pumps. No genes encoding a respiratory Ech (energy-converting hydrogenase), as involved in energy conservation in *Th. phaeum* and *S. schinkii,* were identified in *C. ultunense* and *P. lettingae*. Moreover, two respiratory complexes sharing similarities to the proton-translocating ferredoxin:NAD^+^ oxidoreductase (Rnf) and the Na^+^ pumping NADH:quinone hydrogenase (NQR) were predicted. These might form a respiratory chain that is involved in the reduction of electron acceptors rather than protons. However, involvement of these complexes in acetate oxidation in *C. ultunense* and *P. lettingae* needs further study. This genome-based comparison provides a solid platform for future meta-proteomics and meta-transcriptomics studies and for metabolic engineering, control, and monitoring of SAOB.

## 1. Introduction

Syntrophic acetate oxidation is essential for the biomethanisation of organic matter in engineered anaerobic digestion (AD) processes, in particular, those that are characterised by high ammonia levels [1,2,3]. Engineered biogas processes are being increasingly used in Europe and worldwide due to its potential to meet environmental and climate-related targets and to secure future energy supplies by reducing the exploitation of finite fossil resources and recycling nutrients and energy. When using organic wastes produced by agriculture, municipalities, and food industry as substrates, the biogas process enables a sustainable and economical recycling of nutrients and energy between urban and rural areas. This multi-functionality of the process reduces the competition for land between food and energy production. However, use of high-energy biomass materials, such as industrial food waste, is still challenging, as the degradation of proteinaceous materials releases high levels of ammonia, which has a direct impact on the prevailing methane production pathway, with serious consequences for process stability and efficiency [1,2,3].

Effective processing of organic matter requires the cooperative activity of microbial food chains. Fermentative microorganisms partially degrade polysaccharides, proteins, sugars, and amino acids to carbon dioxide, hydrogen, and short-chain fatty acids, of which acetate is by far the most abundant. Acetate is cleaved to methane and carbon dioxide by the activity of aceticlastic methanogens, a mechanism that is well studied and occurring in anoxic environments, such as aquatic sediments and terrestrial subsurfaces [4]. However, AD processes operating at high ammonia levels rely on so-called syntrophic acetate-oxidising bacteria (SAOB) [5,6,7,8,9,10,11], since aceticlastic methanogens become inhibited under high ammonia conditions [1,2]. The acetate-converting activity of SAOB strongly depends on the abundant presence of hydrogenotrophic methanogens, which enable the thermodynamically unfavourable acetate oxidation (reaction 1) by immediately consuming the hydrogen or formate produced (reaction 2) [12,13,14]. The very small overall free energy change of ΔG^0^′ −35 kJ mol^−1^ (reaction 3) needs to be shared by two organisms that are doing the work:

CH_3_COO^−^ +H^+^ +2H_2_O → 2CO_2_ + 4H_2_   ΔG^0^′ = +95 kJ per mol rct. (1)

4H_2_ + CO_2_ → CH_4_ + 2H_2_O       ΔG^0^′ = −131 kJ per mol rct. (2)

CH_3_COO^−^ + H^+^ + → CH_4_ + CO_2_     ΔG^0^′ = −35 kJ per mol rct. (3)

Other anaerobic environments, such as rice paddy fields, subtropical lake sediments, oil reservoirs, and nutrient-enriched soils, have also been shown to express syntrophic acetate oxidation (SAO) activity [15,16,17,18,19,20,21]. However, no SAOB have yet been isolated from these environments.

To date, only five SAOB species have been characterised [22,23,24,25,26]. Three of these, *Tepidanaerobacter acetatoxydans*, *Clostridium ultunense,* and *Syntrophaceticus schinkii*, were recovered from mesophilic biogas processes, whereas *Thermacetogenium phaeum* and *Pseudothermotoga lettingae* were isolated from thermophilic biogas facilities. Pure culture and enzymatic studies affiliated all but *P. lettingae* to the physiological group of acetogens, producing acetate as the main end product by employing the reductive Wood-Ljungdahl (WL) pathway when growing heterotrophically [3,13,23,25,26,27]. Whole cell extract-based enzyme activities and messenger RNA (mRNA) expression studies indicated that the SAOB use a reversal of the WL pathway for oxidising acetate to hydrogen and carbon dioxide [13,27,28,29]. The thermophilic *P. lettingae* does not express acetogenic metabolism [22], and it must therefore use an alternative pathway. *T. acetatoxydans* might also use another metabolic route for acetate oxidation, as genome-scale analysis has revealed a lack of F_0_/F_1_-ATP (Adenosine triphosphate) synthase, which makes use of the oxidative WL pathway for acetate oxidation energetically unfeasible [30]. In contrast, transcriptomic studies of a methane-producing syntrophic co-culture have demonstrated the expression of the WL pathway genes in *S. schinkii* [28]. Further, genome-based analysis, which has been thoroughly performed for *T. acetatoxydans*, *Th. phaeum,* and *S. schinkii*, indicates different strategies for energy conservation [29,31,32].

Here, we describe genome-based analysis of the mesophilic SAOB *C. ultunense* strain Esp and strain BS^T^, which have recently been sequenced [33,34]. We also compare and summarise selected metabolic features and energy-conserving systems of all known SAOB (*T. acetatoxydans*, *S. schinkii*, *Th. phaeum*, *P. lettingae,* and *C. ultunense*). Genome-guided analyses reported for *Th. phaeum* [32], *T. acetatoxydans* [30] and *S. schinkii* [28] provided the basis for the comparison. In the case of *P. lettingae*, we analysed a publically available genome draft.

The knowledge gained in this study will form the basis for future metabolic engineering and synthetic biology in order to employ SAOB as microbial cellular factories for hydrogen and methane production on demand in a controlled and monitored manner. In particular, when using complex conditions, such as organic waste streams as fuel for microbial anodes, a deep understanding of the physiological attributes of potential electroactive bacteria, such as the SAOB, is needed, in order to achieve successful deployment under competitive industrial conditions.

## 2. Experimental Procedures

### 2.1. Growth Conditions

Since its isolation, *C. ultunense* strain Esp (JCM accession number JCM 16670) has been kept as liquid culture in our laboratory. For DNA isolation, cells were transferred to fresh basal medium. Stock solutions and basal medium were prepared, as described by Zehnder et al. [35]. In brief, stock solutions for basal medium A (15 mL), B (15 mL), I (5 mL), F (1 mL), and 0.2 g/L yeast extract were mixed with 1100 mL distilled water and cooked down to a final volume of 900 mL. The medium was cooled and aliquoted under flushing with N_2_/CO_2_ (80/20, v/v) to 180 mL portions in 500 mL bottles, sealed with butyl rubber stoppers, screw-topped, and autoclaved for 20 min at 121 °C. The cooled bottles were each supplemented with 10 mL of mixture C1 (containing stock solutions E (1mL, trace elements), G (1 mL, vitamin solution), C (12.5 mL) and 34.5 mL distilled water), 10 mL of mixture C2 (containing stock solutions D (49 mL), H (1 mL), and 0.5 g cysteine-HCl) and 2 mL Na-lactate (1 M) as carbon source, by injecting the mixtures and the substrate directly through a sterile filter (0.2 µm) into the bottles. Final pH of the medium was within the neutral range when checked by pH indicator paper. Bottles were inoculated 1:100 with *C. ultunese* strain Esp and harvested at 5000× *g* after four weeks of growth at 37 °C without shaking.

### 2.2. Isolation of DNA

DNA was isolated from 400 mL fresh culture after four weeks of cultivation. The Blood&Tissue Kit (Qiagen, Hilden, Germany) was used for DNA isolation following the standard protocol provided by the manufacturer. The quality was visualised by agarose gel electrophoresis and the quantity determined by fluorometric measurements using Qubit (Thermo Fisher Scientific, Waltham, MA, USA).

### 2.3. Genome Sequencing and Assembly

*C. ultunense* strain Esp was sequenced at the SciLife Laboratory (Uppsala, Sweden). The genome was first sequenced using the Ion Torrent PM system (Thermo Fisher Scientific, https://www.thermofisher.com) with a de novo assembly approach, which produced 281 scaffolds with a total size of 6,159,766 base pairs (bp). Sequencing and assembling details are reported in [34]. In order to improve the quality of the genome, we used HiSeq-2500 mate-pair sequencing (Illumina, https://www.illumina.com) with a library size of 3 Kb in mapping assembly.

Read length of 206 bp, a longest read length of 392 bp, and a total of final library reads of 2,631,078 for single end reads were obtained from Ion Torrent sequencing and 82,386,171 from the Illumina mate pair sequencing. General details about the sequencing performed can be found on the SciLifeLab website [36]. The FastQC software package [37] was used for read quality assessment. After pre-assembly quality checking, the filtered reads were fed into MIRA 4.0 [38] assembler for both mapping and de novo assembly, and the same reads data were also provided to the Newbler 2.8 de novo assembler (Roche, http://www.my454.com). Mapping assembly was undertaken against the available genome of *C. ultunense* strain BS (DSMZ 10521) (accession No. AZSU00000000) [33]. Four different assemblies were produced: (i) de novo assembly of Ion Torrent reads; (ii) de novo assembly of Illumina reads; (iii) hybrid de novo assembly of Ion Torrent reads and Illumina reads; and, (iv) hybrid mapping assembly of Ion Torrent reads and Illumina reads. Resulting assemblies were compared using the Mauve genome alignment tool [39]. However, during this process, we discovered that the culture used for DNA purification was impure. The impurity could be identified as *Thermicanus aegyptius*, which draft genome sequence is publically available (BioProject: PRJNA81927, RefSeq: NZ_AZU00000000.1, total length: 3.657 Mb). Thus, the hybrid mapping assembly produced from Ion Torrent reads and Illumina reads was selected for further analysis. Selection was based on N50 statistics, number of contigs, and the length of the largest contig. Scaffolding of the selected assembly was performed using SSPACE [40] and the removal of possible gaps present in scaffolds was performed using GapFiller [41]. Homopolymer errors were corrected manually using Consed [42]. The purity of the scaffolds was also checked against the genome of *T. aegyptius* DSM 12793. Possible miss-assemblies were corrected manually using Tablet, a graphical viewer for the visualisation of assemblies and read mappings [43]. Based on these analyses, the high-quality permanent genome that was obtained was only half the size (3,093,245 bp) of that sequenced by the Ion Torrent PM system [34]. The genome project is deposited in the Genomes On Line Database [44] with GOLD id Gp0161004, and the genome sequence is deposited in the European Nucleotide Archive database with accession number ERS1433212. Genome statistics are summarized in Table S1. Due to the bioinformatics approach that is applied, we do not expect any contamination of the high-quality draft genome sequence however we cannot entirely exclude incompleteness of the genome.

### 2.4. Genome Annotation and Genome Comparison

Automated gene modelling was performed using the Microbial Genome Annotation & Analysis Platform MaGe [45], a bacterial genome annotation system. Genes were identified using Prodigal [46] and AMIGene [47] as part of the MaGe genome annotation pipeline. The predicted CDSs (coding sequences) were translated and were used to search the National Center for Biotechnology Information (NCBI) non-redundant database and UniProt, TIGRFam, Pfam, PRIAM, Kyoto Encyclopedia of Genes and Genomes (KEGG), Clusters of Orthologous Groups (COG), and InterPro databases using Basic Local Alignment Search Tool Protein (BLASTP) (https://blast.ncbi.nlm.nih.gov/). Predicted coding sequences were subjected to manual analysis using the MaGe web-based platform, which also provides functional information about proteins and was used to assess and correct genes predicted through the automated pipeline. The predicted functions were also further analysed by the MaGe annotation system. Genomic features of *C. ultunense* strain Esp, *C. ultunense* strain BS^T^ (accession number AZSU00000000), *T. acetatoxydans* strain Re1 (accession number HF563609), *S. schinkii* strain Sp3 (accession number CDRZ00000000), *Th. phaeum* strain PB (accession number CP003732), and *P. lettingae* strain TMO (accession number CP000812.1) were compared using the MaGe web-based platform. Prophage sequences were identified using the PHASTER web server [48]. Tandem duplicated genomic regions of protein coding genes were calculated on the basis of identity (≥35%), minLRap (≥0.8) and gene separation (maximum of five consecutive genes) by using (MaGe) [45].

### 2.5. Phylogenetic Placement and Synteny

The 16S ribosomal DNA (rDNA) sequence alignment was carried out using MUSCLE [49] and a phylogenetic tree was constructed using the maximum-likelihood (ML) algorithm [50] with MEGA 6.06 [51,52]. Bootstrap analysis [53] with 100 replicates was performed to assess the support of the clusters. All predicted gene loci from *C. ultunense* strain Esp were subjected to linear comparisons with *C. ultunense* strain BS^T^, *S. schinkii*, *T. acetatoxydans*, *Th. phaeum*, and *P. lettingae*, using the built-in tool in the MaGe platform with synton size ≥ 3 genes.

## 3. Results

### 3.1. Phylogenetic Placement

**Figure 1:**
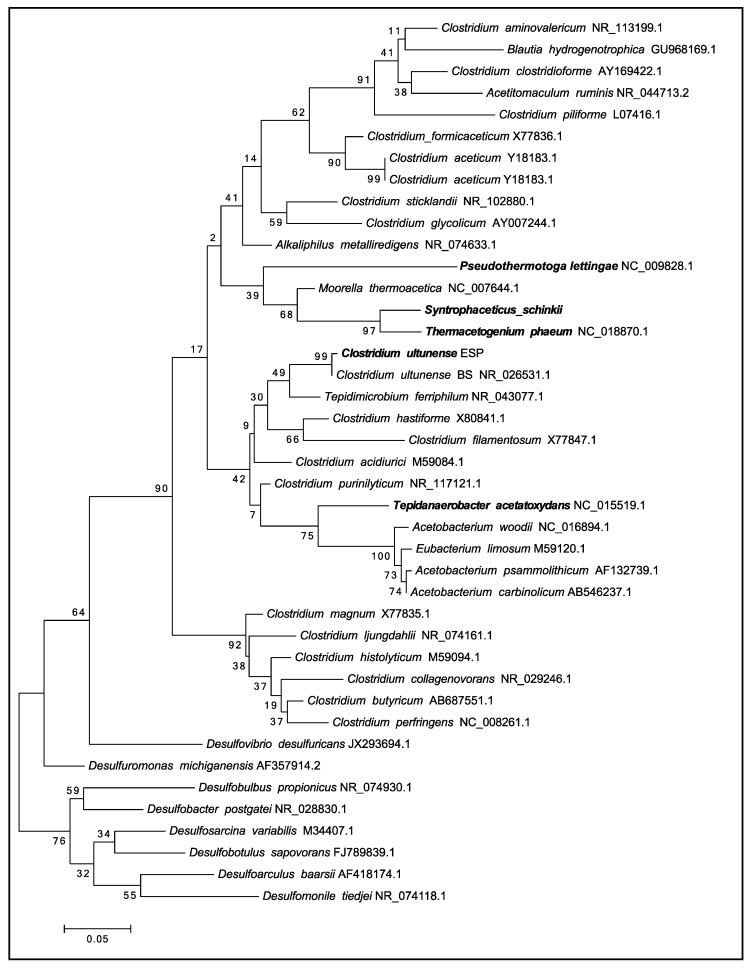
Maximum likelihood tree of the 16S ribosomal RNA (rRNA) gene showing the phylogenetic position of the syntrophic acetate-oxidising bacteria (SAOB) *Clostridium ultunense*, *Syntrophaceticus schinkii*, *Tepidanaerobacter acetatoxydans*, *Pseudothermotoga lettingae*, and *Thermacetogenium phaeum* related to acetogens and sulphate reducers.

Phylogenetic analysis of the single 16S rDNA affiliates *C. ultunense* strain Esp to the Clostridia class within the phylum Firmicutes, and therein to the family Tissierellaceae [54] (Ribosomal data base project (RDP Naive Bayesian rRNA Classifier Version 2.11, 2017-12-11). The 16S rDNA gene of the type strain BS^T^ (=DSM 10521) is 99% identical to strain Esp (Figure 1). Comparison of the 16S rDNA gene against the latest available databases from GenBank (2018-01-22) using BLAST under default settings shows that *Clostridium* sp. MT1 isolated from the human gut (LK021112) is the closest current relative, sharing 97% identity. The closest characterised species is *Keratinibaculum paraultunense*, sharing 95.3% identity [55]. *K. paraultunense* was isolated from grassy marshland and it has been characterised as a thermophilic, anaerobic, keranolytic bacterium. The model acetogens *Acetobacterium woodii* and *Moorella thermoacetica* share 85% and 83% 16S rDNA gene identity, respectively (Figure 1). *C. ultunense* strain Esp is also only distantly related to the recently characterised mesophilic SAOB *S. schinkii* (83% identity) and *T. acetatoxydans* (82% identity), and to the thermophilic SAOB *P. lettingae* (76%) and *Th. phaeum* (85%) (Figure 1).

Synteny-based analysis revealed that *C. ultunense* strains BS^T^ and Esp have approximately 98% of the total genome size in synteny (Figure S1). Synteny-based analysis with all bacterial genomes that were present in the NCBI database reference sequence (RefSeq) revealed that *Alkaliphilus metalliredigens QYMF* appeared to be the closest current relative, with approximately 43% of its total genome size in synteny with *C. ultunense* strain Esp (Figure S2). Likewise, a comparison of all the inferred proteins of *C. ultunense* strain Esp with all proteins collected in the NCBI RefSeq database revealed the highest number of orthologues with *C. ultunense* strain BS^T^ (3106: 91.87%) and the next highest with *A. metalliredigens* QYMF (1920: 56.79%).

**Figure 2:**
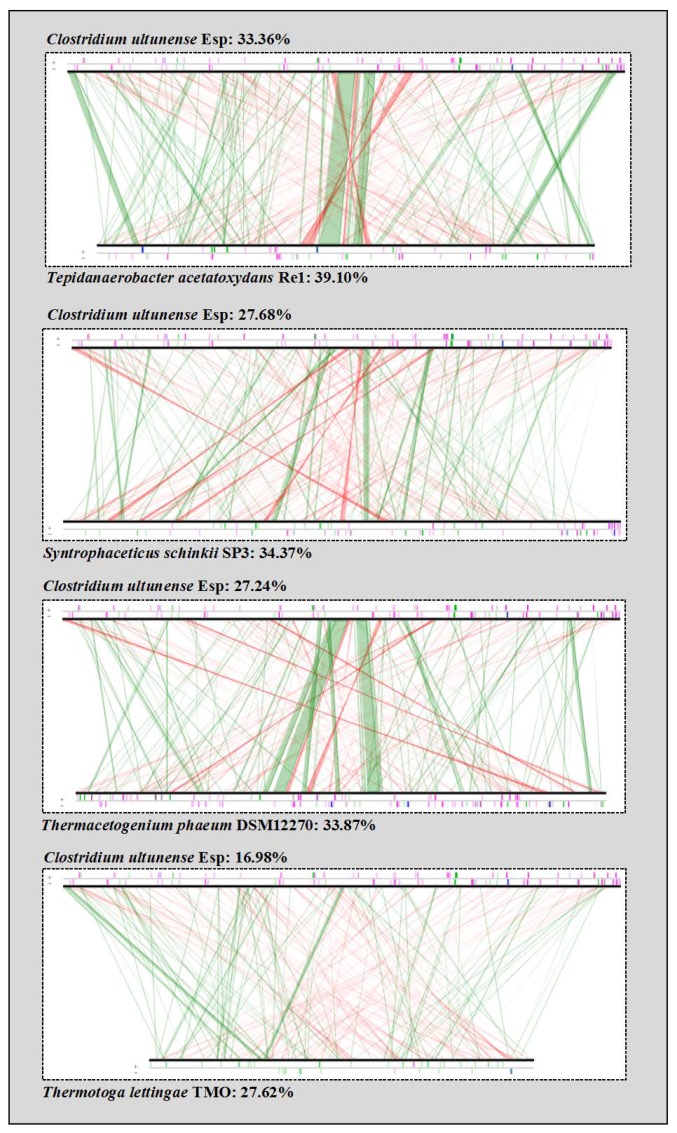
Synteny comparison of C. ultunense strain Esp and the SAOB *S. schinkii*, *T. acetatoxydans*, *Th. phaeum*, and *P. lettingae.* The lines indicate syntons between two genomes. Red lines show inversions around the origin of replication. Vertical bars on the border line indicate different elements in genomes, where pink = transposases, or insertion sequences, blue = rRNA and green = transfer RNA (tRNA).

In general, synteny analysis and comparison of inferred proteins showed a lower relationship of *C. ultunense* strain Esp to other SAOB than to *A. metalliredigens* (Table 1, Figure 2).

### 3.2. Genome Properties

The draft genome sequence for *C. ultunense* strain Esp comprises one scaffold with a total size of 3,093,245 bp and a calculated GC content of 32.40%. The genome showed a protein coding density of 97.97%, with an average intergenic length of 130.04 bp. The genome contains 66 tRNA, four 5S, one 16S, and one 23S rRNA genes (Table S1, Figure S3). No tRNA for selenocysteine incorporation was affiliated automatically by the MaGe pipeline. However, the genome harbours genes for *L*-selenocysteinyl-tRNA Sec transferase (selA: CUESP_2577, AZSU01_50111), monoselenophosphate synthase (selD: CUESP_2578, AZSU01_50112), and selenocysteinyl-tRNA elongation factor (selB: CUESP_2576, AZSU01_50110), as observed also for *S. schinkii* [31] and *Th. phaeum* [32]. For both *S. schinkii* and *Th. phaeum*, a selenocysteine-tRNA was annotated. However, selenocysteine-containing proteins are only predicted for *C. ultunense* Esp/BS^T^ and *S. schinkii* (glycine/betaine reductase complex: CUESP_0484-90, CUESP_02409-11, CUESP_0601-03; AZS01_10478-84, _40044-46, _20010-12 [34]). The genome of *C. ultunense* strain Esp contains 3303 predicted protein-encoding genes, of which 2198 (66.54%) have been assigned tentative functions. The remaining 1105 ORFs (open reading frames) are hypothetical/unknown proteins. It was possible to allocate 2623 (app. 79.41%) of all the predicted protein-encoding genes to the 23 functional COGs. That number was slightly higher than predicted for *C. ultunense* strain BS^T^ (2594 genes; 77.78%; 21 COGs). In all, 33.7% of the protein-encoding genes fell into the metabolism category, within which they were mainly affiliated to: energy metabolism (5.54%), amino acid transport and metabolism (9.23%), carbohydrate transport and metabolism (5.26%), and inorganic ion transport and metabolism (4.60%) (Table 2). These values are within the range observed for the other SAOB, where carbohydrate metabolism shows the widest range.

Cultivation experiments indicated that *C. ultunense*, *S. schinkii,* and *Th. phaeum* are very confined in heterotrophic substrate utilisation, in the case of *C. ultunense* strain Esp encompassing only ethanol, betaine, lactate, cysteine, and raffinose [23,24,25]. *P. lettingae* instead can use a much broader substrate spectrum [22] and *T. acetatoxydans* also proved somewhat more versatile [26], both showing higher percentages in this COG category (Table 2).

Both *C. ultunense* genomes (Esp and BS^T^) were found to harbour several CRISPR (Clustered Regularly Interspaced Short Palindromic Repeats) loci. The genomes of strain Esp and BS^T^ each harbour one operon for encoding cas proteins (CUESP_2343-2350, AZSU01_30868-30875) and six to nine CRISPR loci were predicted. In *T. acetatoxydans*, *Th. phaeum,* and *S. schinkii*, two operons each have been identified and more than eight CRISPR loci each [28,30,32]. The *P. lettingae* genome contains one operon encoding CAS proteins (Tlet_0203-0210) and two CRISPR loci were predicted.

Strain Esp and strain BS^T^ are both lysogens, as their genomes harbour one prophage each. A smaller number of phage remnants were found in strain Esp. In the case of *P. lettingae*, only phage remnants were predicted (Table S2). This is within the range that was observed for the other SAOB (Table S2) [28,30,32]. *T. acetatoxydans* and *Th. phaeum* are lysogens. The prophage of the latter has been shown to be inducible [32]. All together, the genomes of the SAOBs contain between 1.1% and 3.3% prophage-related genes (Table S2).

### 3.3. Morphological and Physiological Traits

*C. ultunense* Esp/BS^T^ was shown to be able to form endospores, which is a trait that is common to all SAOB except *P. lettingae* [22,23,24,25,26]. The master regulator Spo0A needed for sporulation 37 [56] is encoded (CUESP_1786; AZSU01_30330), but the *C. ultunense* Esp/BS^T^ genome lacks genes encoding the phosphorylases (Spo0F, Spo0B), as has been observed in other clostridia 37 [56]. The sporulation-specific sigma factors SigE, SigG, SigF, and SigA were identified (CUESP_1917-18, AZSU01_30452-53; CUESP_1969; AZSU01_30503; CUESP_2058, AZSU01_30586).

The genome encodes both flagellum-related and chemotaxis-related genes (CUESP_1643-1678; AZSU01_30193-30220), which is in accordance with previous observations that *C. ultunense* Esp/BS^T^ possesses a polar flagellum and can perform tumbling movements [24,25]. A similar genotype and phenotype have been described for *T. acetatoxydans* [30], *Th. phaeum* [32], and *P. lettingae* ([22] (Tlet_0622-_0629; 0079-_0081; 1898-_1901; 1819-1826). In contrast, *S. schinkii* appears to be the only SAOB that lacks the ability to perform chemotactic manoeuvres [28].

*C. ultunense* Esp/BS^T^ can potentially tolerate oxidative stress, as the genome encodes rubrerythrin (AZSU_10385, CUESP_0386), which can reduce hydrogen peroxide (H_2_O_2_) to water. Rubrerythrin encoding genes were also reported for *S. schinkii*, *Th. phaeum* and *T. acetatoxydans* [28,30,31,32]. To protect against superoxide radicals (O_2_^−^), *C. ultunense* Esp/BS^T^ may express superoxide dismutase (CUESP_0551, _02564; AZSU01_10540, _50098) and superoxide reductase (CUESP_3088; AZSU01_70055). The genome of *P. lettingae* also encodes rubrerythrin (Tlet_0923) and superoxide reductase (Tlet_1498).

A characteristic of the mesophilic SAOB is their high ammonia tolerance. Potential mechanisms in *C. ultunense* Esp/BS^T^ preventing NH^4+^/NH^3-^ induced osmotic stress might include putative potassium uptake proteins (CUESP_3204-3205; CUESP_2895-2896), as predicted for *S. schinkii* [28] and *T. acetatoxydans* [30]. Homologues have been also predicted for the genomes of *P. lettingae* (Tlet_0892-0893) and *Th. phaeum*: (Tph_28930-28960-2, Tph_8850-28840). Potential betaine/glycine transport systems in order to accumulate compatible solute, as found in the genomes of *Th. phaeum*, *S. schinkii* [28], and *T. acetatoxydans* [30], are not predicted for *C. ultunense* Esp/BS^T^ or *P. lettingae*. However, similarly to *T. acetatoxydans* [30] and *S. schinkii* [28], no gene encoding high ammonium affinity glutamine synthetase (GS) was found in the *C. ultunense* Esp/BS^T^ genome. Instead, the low ammonium affinity glutamate dehydrogenase (GDH) pathway appears to play a major assimilatory role (CUESP_1377; ASU01_20769). In contrast, the genomes of the thermophilic SAOB *Th. phaeum* and *P. lettingae* harbour homologues to GS (Tph_12990, 08620, 09680, 12980; Tlet_0896, _2058) and GDH (Tph_04750; Tlet_0263). Among the SAOBs that were investigated, putative ammonium transporters were only predicted for *Th. phaeum* (Tph_08750). V-type ATPases suggested to support *T. acetatoxydans* in maintaining pH homeostasis [30] were not found in *C. ultunense* Esp/BS^T^ or in the remaining SAOB.

*C. ultunense* strain Esp harbours about 33 ABC transport systems, of which 11 are predicted to transport oligopeptides and amino acids, which is in agreement with the observed requirement for yeast supplement for growth [24,25]. Five ABC transport systems likely shuffle ribose and guanosine over the membrane, while two transport Fe and Co, respectively (Table S3). Likewise, the majority of the ABC transport systems found encoded by the genomes of *P. lettingae* (Table S4) and *T. acetatoxydans* [30] are predicted to transport amino acids and oligopeptides (Table S4). In *Th. phaeum* and *S. schinkii*, ABC transport systems are mainly predicted to transport trace elements that are needed as cofactors [28] (Table S5), rather than organic compounds. There are at least four phosphoenolpyruvate (PEP) carbohydrate phosphotransferase systems (PTS) that are encoded in the *C. ultunense* strain Esp genome. These are predicted to take up bacterial cell wall components, such as N-acetylglucosamine and N-acetylmuramic acids and oligocarbohydrates potentially originating from lichenan or related polymers (Table S6). The general PTS proteins Hpr and EI (CUESP_0086, _0088) and Hpr kinase (CUESP_0224, AZSU_10221) and Hpr-like protein (AZSU_10234, _20320; CUESP_0236, _0914) that are needed for CcpA-dependent regulation of carbon metabolism [57] are encoded elsewhere. Genomic evidence for PTS transport systems and carbon catabolite repression/activation has only been found in *T. acetatoxydans* [30], and not in *S. schinkii* [28], *Th. phaeum* or *P. lettingae*. One potential TRAP transporter (CUESP_2539-2540) using the ion gradient over the membrane to energise unidirectional transportation of solutes was found encoded in the *C. ultunense* strain Esp genome. TRAP transporters are only predicted for the genome of *T. acetatoxydans* [30] and *P. lettingae* (Tlet_0052-0054), and not for *Th. phaeum* and *S. schinkii* [28]. Putative acetate uptake transporters are only predicted for *S. schinkii* and *Th. phaeum* (Table S6) [28,32].

### 3.4. Wood-Ljungdahl Pathway and Acetate Activation

One of the central activities of acetogenic metabolism is mediated by phosphotransacetylase (PTA), which replaces the CoA moiety from acetyl-CoA by an inorganic phosphate molecule to form acetylphosphate. The phosphate moiety is further transferred by the activity of acetate kinase (AckA) to adenosine diphosphate (ADP), producing ATP and the end product acetate. The *C. ultunense* Esp/BS^T^ genome harbours an *ackA* gene (CUESP_1616, AZSU01_30167), but it lacks a *pta* gene. Thus, substrate-level phosphorylation through Pta/AckA is strongly impaired. Moreover, only genes encoding proteins of the methyl branch of the WL pathway are present. These include formyl tetrahydrofolate (THF) synthetase (product of CUESP_1461; AZSU01_20850), bifunctional methylene THF dehydrogenase/methenyl THF cyclohydrolase (product of CUESP_1972, AZSU01_30506), and formate dehydrogenase (CUESP_2852-2856; AZSU01_60163-60167). The first two proteins are widely distributed in bacteria, as their activities provide the cells with one-carbon donors at different oxidation levels for biosynthesis. Two potential carbon monoxide dehydrogenases (CODH) were predicted elsewhere: One together in an operon with genes encoding a ferredoxin-binding subunit and an NAD(P)-dependent oxidoreductase (AZSU01_20423-25; CUESP_1023-25); and, the other consisting of two subunits and forming a potential operon with a membrane protein of unknown function (CUESP_2144-47, AZSU01_30669-72). According to synteny maps obtained with genomes of acetogens, neither of those resemble the CODH subunit of the acetyl-CoA/CODH complex as part of the WL pathway. Moreover, the essential CODH-Ni-insertion protein (CooC) is lacking. Genes encoding acetyl CoA synthase subunit, methyltransferase, methylene reductase, and the corrinoid enzyme are absent. Despite this, acetogenic metabolism and acetyl-CoA synthase activities have been reported for the *C. ultunense* strain BS^T^ [13,24]. To achieve this, Pta/AckA activity could potentially be replaced by a predicted ADP-forming Acetyl-CoA synthetase (CUESP_2312, AZSU01_30838). Alternatively, side activities of other phosphate acyltransferases (e.g., CUESP_0095; AZSU_10093; CUESP_1619, AZSU01_30169; CUESP_1223, AZSU01_20621) might substitute for the function of PTA. Enzyme activity measurements that were performed previously [13] are not specific for PTA, because the release of free CoASH from acetyl-CoA is determined [58,59]. Thus, free CoASH can be produced by any acyl-CoA active enzyme, which might explain the observed activity of the cell-free extract [13]. Consequently, the reported weak growth on lactate, pyruvate, ethanol, and *L*-cysteine must either rely on the abovementioned activities of either ADP-forming Acetyl-CoA synthetase or phosphate acyltransferases. Alternatively, the final product acetyl-CoA is used in anabolic pathways, rather than in energy conservation, and the actual energy source is provided by the added yeast extract. A malate/lactate dehydrogenase-like protein (CUESP_3146), alcohol, and aldehyde dehydrogenase (CUESP_1073-76, AZSU01_20472-74), Pyruvate:ferredoxin/flavodoxin oxidoreductases (CUESP_2474/75; AZSU01_50009/10; CUESP_2527, _0225; AZSU01_50061, _10222), and *L*-cysteine disulphydrase (CUESP_2922, _2143) are encoded by the *C. ultunense* Esp/BS^T^ genome.

In contrast, the genomes of *S. schinkii*, *T. acetatoxydans*, and *Th. phaeum* harbour the WL pathway genes as well as genes encoding phosphotransacetylase and acetate kinase [28,30,32], which is in accordance with the acetogenic metabolism that is observed in pure cultures. *P. lettingae*, in which growth has been described as non-acetogenic [22], does not harbour genes that are related to phosphotransacetylase, acetyl-CoA synthetase, ADP-forming acetyl-CoA synthetase, carbon monoxide dehydrogenase, methyltransferase, methylene THF reductase, or format dehydrogenase/lyase. However, genes for acetate kinase (Tlet_0293), formyl THF synthetase (Tlet_1513), and bifunctional methylene THF dehydrogenase/methenyl cyclohydrolase/(Tlet_1514) are encoded.

### 3.5. Respiratory Complexes

**Figure 3:**
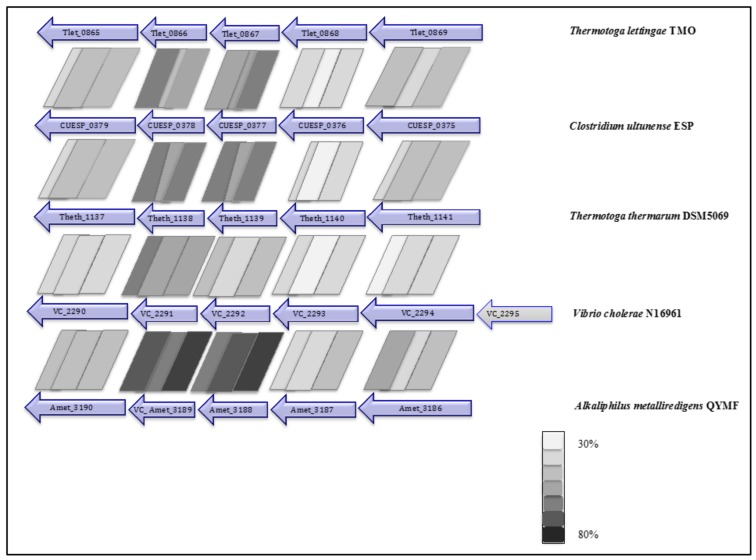
Organisation of the *Nqr* genes in *C. ultunense*, *P. lettingae*, *Pseudothermotoga thermarum*, *Vibrio cholera*, and *Alkaliphilus metalliredigens*.

Two potential ion motive electron transport complexes were identified in the genome of *C. ultunense* strain Esp (Table 3). An operon encodes a protein complex (CUESP_0375-0379; AZSU01_10374-10378), which shows similarity to the Na^+^ pumping NADH:quinone hydrogenase (Na^+^-NQR), which is a respiratory complex that is widely distributed in marine bacteria, which couples electron transport to the generation of a sodium gradient [60]. It is composed of six subunits (NQRA-F) pumping out Na^+^, while electrons travel from reduced nicotinamide adenine dinucleotide (NADH) to quinone via a minimum of five redox carriers [60,61]: one Flavin adenine dinucleotide FAD and one 2Fe-2S cluster in the peripheral NQRF subunit, one flavin mononucleotide (FMN) in the hydrophilic NQRC subunit, and one FMN and riboflavin in the membrane integral NQRB subunit. NQRF is the site for NADH oxidation and NQRA has been identified as the quinol formation site. The complex has also been identified in the genome of the SAOB *P. lettingae* (Tlet_0865-0869, Table 3) and its close relative *Pseudothermotoga thermarum* [62]. The genes are arranged in the order *NqrBCDEF* and share between 36% and 55% identity (Figure 3). However, the genome lacks a NQRA subunit gene that contains the quinone binding site, as has also been reported for *P. lettingae* and *P. thermarum* [62]. The genomes of *T. acetatoxydans*, *Th. phaeum,* and *S. schinkii* do not encode any *Nqr* genes [28,30,32].

As found in *T. acetatoxydans* [30] and *S. schinkii* [28], the genomes of *C. ultunese* Esp/BS^T^ (AZSU01_50145-50150; CUESP_2612-2617) and *P. lettingae* (Tlet_0286-0291), contain a gene cluster each coding for the respiratory Rnf complex. The Rnf complex is a ferredoxin:NAD^+^ oxidoreductase that uses the redox span between ferredoxin (E′ = −500 mV) and NADH (E′ = −320 mV) to establish an ion gradient over the membrane [63]. This is equivalent to one-third to one-half of an ATP. Conversely, the generated Δμ˜_Na_^+^_/H_^+^, can drive the endergonic electron flow from NADH to ferredoxin. The genes are organised in the order *rnfCDGEAB*, as found in many Clostridia [63]. The genome of *Th. phaeum* lacks Rnf genes [32] (Table 3).

The genome of *C. ultunese* Esp/BS^T^ does not harbour genes that are related to Ech (energy-converting hydrogenase) hydrogenase that have been found in *S. schinkii* and *Th. phaeum* [28,32]. Likewise, the genomes of *T. acetatoxydans* [30] and *P. lettingae* do not encode Ech hydrogenase (Table 3). The Ech hydrogenases belong to the group of [Ni-Fe] hydrogenases, which catalyse the reversible exergonic oxidation of reduced ferredoxin to the reduction of protons that are coupled to vectoral ion transport across the membrane [64].

### 3.6. Soluble Electron Transfer Proteins

The genome of *C. ultunense* Esp/BS^T^ encodes several ferredoxins, one rubredoxin, and flavodoxins (Table 3). A similar composition of soluble electron transfer proteins has been found for the genomes of *P. lettingae* and *Th. phaeum*, including flavodoxins, ferredoxins, and rubredoxin (Table 3). However, the genomes of *S. schinkii* do not harbour any flavodoxin-related genes [28,30,32] (Table 3). Of all SAOB genomes, only *P. lettingae* encodes a cytochrome (Table 3). One electron transfer flavoprotein (ETF) complex was found to be encoded in the genome of *C. ultunense* Esp/BS^T^ (*Etf*A,B CUESP_1366-1368, AZSU01_20759-20761). The genomes of *P. lettingae* (Tlet_1692-1693; Tlet_1832-1833), and *T. acetatoxydans* (TepiRe1_2532-2533) were also found to harbour ETF complex genes. No ETF-related genes were identified in the genomes of *Th. phaeum* and *S. schinkii* (Table 3).

### 3.7. Hydrogenases

The genome of *C. ultunense* Esp/BS^T^ encodes one putative [Fe-Fe] hydrogenase (CUESP_2849-2850; AZSU_01_60160-60162) that is adjacent to genes coding for a cytoplasmatic formate dehydrogenase (CUESP_2852-2856; AZSU01_60163-60167). The short intergenic regions indicate a potential operon. A similar gene organisation is found in the genome of *Th. phaeum* (Tph_c18420-_c18430) [32] (Table 3). Putative [Fe-Fe] hydrogenases have also been identified in the genomes of *T. acetatoxydans* [30], *S. schinkii* [28], and *P. lettingae* (Tlet_0952-0957; Tlet_1518-1522), with *S. schinkii* harbouring four duplicates, the highest number. In contrast to *T. acetatoxydans* and *P. lettingae*, the genome of *S. schinkii* contains both cytoplasmatic and membrane-bound formate hydrogenases, but these are located separately from any hydrogenase-related genes [28] (Table 3). The genome of *C. ultunense* Esp/BS^T^, and the genomes of *T. acetatoxydans* and *P. lettingae*, do not contain genes that are related to [Ni-Fe] hydrogenases. However, those complexes have been found in the genome of both *S. schinkii* and *Th. phaeum* (see [64] for classification of hydrogenases). Genes that were predicted to encode a putative NAD(P)-binding oxidoreductase/heterodisulphide reductase complex and a putative formate lyase complex seem to be unique to the genomes of *S. schinkii* and *Th. phaeum* [28,32], respectively, as no such complexes are predicted for the genomes of *C. ultunense* Esp/BS^T^, *T. acetatoxydans,* and *P. lettingae* (Table 3).

### 3.8. Adenosine Triphosphate (ATP) Synthase and Pyrophosphatase (PPase)

Next to the ATP synthase complex (CUESP_1228-1235; AZSU_20626-20633), the genome of *C. ultunense* Esp/BS^T^ encodes a putative membrane-bound ion translocating pyrophosphatase (CUESP_0504; AZSU01__10499). ATP synthase and putative pyrophosphatase (PPase) genes are also predicted for the genomes of the other SAOB (Table 3), except for *T. acetatoxydans*, the genome of which does not encode an ATP synthase complex [30].

## 4. Discussion

### 4.1. Attributes Linked to Anaerobic Digestion Environments

It has been shown repeatedly that the presence and abundance of SAOB in engineered methanogenic processes are strongly positively correlated with high ammonia concentrations. Factors such as acetate concentration, dilution rate, and methanogenic community structure have also been suggested to promote SAO activity [5,6,7,8,9,10,65,66,67,68,69,70,71,72]. However, certain competitive abilities can be accredited to *S. schinkii*, as this SAOB has been found at high abundance under both low- and high-ammonia conditions [9,10,68,73]. Potential competiveness for the substrate acetate has been suggested as one explanation, as *S. schinkii* expresses a putative acetate uptake system [28], which is otherwise only encoded by the genome of the thermophilic *Th. phaeum*.

Another factor affecting the abundance of SAOB might be the proportion of proteinaceous material that is provided in the feedstock, as indicated by the genomic equipment of SAOB. A large proportion of the transport systems in all SAOB are predicted to shuffle oligopeptides and amino acids over the membrane, rather than carbohydrates. Furthermore, a number of genes that are needed for de novo synthesis of amino acids are lacking, which explains the observed dependency on yeast extract when cultivating SAOB under laboratory conditions. Thus, higher availability of proteins in the feedstock might be beneficial, as it provides both the precursors for biosynthesis and energy sources. Apart from *Th. phaeum*, none of the SAOB harbours genes for ammonium transporters and the mesophilic SAOB also lack the high ammonium affinity GS, both further emphasising an adaptation to amino acid-rich environments. At this time, there are no indications of habitats other than engineered AD processes when searching for 16S rRNA gene traces in the nucleotide (nr/nt) database. *C. ultunense* Esp/BS^T^ has been identified in a fermenting woad vat, which is another artificial environment [74]. However, all SAOB are spore-forming, which enables (re)-entry and (re)-population of biogas processes depleted in SAOB, and is beneficial when conditions become lethal, e.g., through exposure to oxygen. Moreover, none of the known SAOBs appears to be a strictly obligate anaerobe, as indicated by genes conferring protection against super oxide radicals. This attribute facilitates the transfer between cultivation bottles and bio-augmentation of laboratory-scale reactors, as well as phenotypic characterisation and metabolic engineering.

One more interesting attribute of the SAOB is their apparent robustness against phages. In nutrient-rich and cell-dense environments such as AD processes, viruses can be expected to be high in numbers and diversity [75]. Despite the high virus abundance in AD environments, the genomes of *C. ultunense* Esp/BS^T^ and other SAOB appear to be only weakly infected. This indicates a certain robustness against phage attacks, as bacterial genomes can contain up to 20% prophage-related genes [76,77]. The significantly higher number of CRISPR loci acquired by the SAOB points to strong phage-microbial interactions. CRISPRs have been found in almost half of all the bacterial genomes and almost all of the archaeal genomes sequenced [78]. The cas/CRISPR system is a prokaryotic defence mechanism, enabling the organism to respond to and eliminate invading genetic material, such as bacteriophages [79]. The CRISPR-associated sequence (cas) genes are often directly adjacent to the CRISPR loci. The number of CRISPR loci ranges from one to 21, but up to three loci occur most frequently [32]. The adaptive nature of CRISPR triggered by invading phages confers robustness and the ability to encounter new phages. Thus, the strong impact of bacteriophages on microbial diversity and community composition in AD processes, as recently shown by Zhang et al. [75], might be less pronounced in SAOB populations. There are also indications that phages selectively attack the larger populations within a microbial community [80,81]. Moreover, the CRISPR/cas loci that were identified in SAOB might potentially enable strain-typing applications for controlling naturally and metabolically engineered SAOB strains.

### 4.2. Acetate Oxidation in Mesophilic and Thermophilic Syntrophic Acetate-Oxidising Bacteria

**Figure 4:**
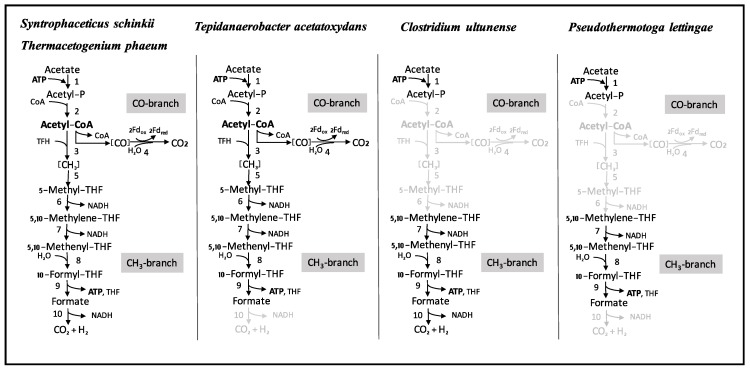
Wood-Ljungdahl (WL) pathway proteins encoded by the genomes of the SAOB *S. schinkii*, *Th. phaeum*, *T. acetatoxydans*, *C. ultunense* Esp/BS^T^, and *P. lettingae*. (1) Acetate kinase, (2) phosphotransacetylase, (3) acetyl CoA synthetase complex (including corrinoid protein), (4) carbon monoxide dehydrogenase, (5) methyl transferase, (6) methylene THF reductase, (7) methylene tetrahydrofolate (THF) dehydrogenase, (8) methenyl THF cyclohydrolase, (9) formyl THF synthetase, (10) formate dehydrogenase. Functions displayed in light grey are not encoded.

**Figure 5:**
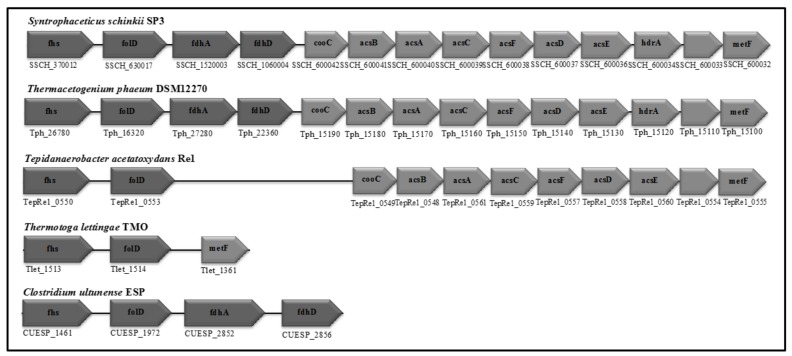
Organisation of the WL pathway genes in the SAOB *S. schinkii*, *Th. phaeum*, *T. acetatoxydans*, *C. ultunense* strain Esp (also valid for strain BS^T^) and *P. lettingae*. acsA,B,C,D,F, Carbon monoxide dehydrogenase/Acetyl-CoA synthetase subunits; acsE, Methyltransferase; hdr, heterodisulphide reductase; metF, methylene tetrahydrofolate reductase; folD, bifunctional methenyl tetrahydrofolate cyclohydrolase/methylene tetrahydrofolate dehydrogenase; fhs, formyl tetrahydrofolate reductase; cooC, accessory protein; fdhA/fdhD, formate dehydrogenase. Light grey: Genes are organized in a putative operon. Dark grey: Genes are scattered in the genome.

Cultivation experiments, enzyme activity studies and mRNA-based investigations previously led to the assumption that *C. ultunense* Esp/BS^T^ belongs to the physiological group of acetogens, indicating that the WL pathway plays a central metabolic role, as has also been reported for the SAOB *Th. phaeum*, *S. schinkii,* and *T. acetatoxydans* [13,24,25,26,29,30,32]. Consequently, in AD processes, these SAOB were thought to be generally able to perform two metabolic functions: acetate production from smaller organic molecules employing the reductive direction of the WL pathway and syntrophic acetate oxidation using the oxidative direction. However, our study revealed that at least two pathways for mesophilic acetate oxidation must exist, as the genome of *C. ultunense* Esp/BS^T^ lacks key enzymes of the WL pathway (Figure 4 and Figure 5).

In the case of the thermophilic *P. lettingae*, the lack of WL pathway genes was expected, as the metabolism has been described as non-acetogenic [22] (Figure 4 and Figure 5). Both *Th. phaeum* and *S. schinkii* harbour the complete set of WL pathway genes (Figure 4 and Figure 5), and transcriptomic profiling of an acetate-oxidising co-culture has clearly confirmed the involvement of the oxidative WL pathway in acetate oxidation in *S. schinkii* [28,32]. For *Th. phaeum*, no such expression data are available, but enzyme activity studies have confirmed expression of key functions of the WL pathway, indicating that this pathway is a potential acetate oxidation route [27]. Moreover, *Th. phaeum* and *S. schinkii* are closely related (92.1% 16s RNA gene identity), with 50% of the genomes in synteny. Gene organisation of the WL pathway genes in *Th. phaeum* is identical to that in *S. schinkii* (Figure 5). Genome-guided analysis of *T. acetatoxydans* has revealed a truncated WL pathway [30] (Figure 4). However, a role of the WL pathway in acetate oxidation has been questioned, as net ATP synthesis is strongly impaired due to the lack of F_0_/F_1_ ATP synthase [30], and substrate level phosphorylation via the oxidative WL pathway does not gain any net ATP (Figure 4). There has been speculation about acetate oxidation in *T. acetatoxydans*, including a metabolic route via an oxidative tricarboxylic acid cycle [30]. However, no evidence on gene expression or activity level has been supplied so far. In the case of *C. ultunense* Esp/BS^T^ and *P. lettingae*, this proposed metabolic route cannot be applied, as both genomes lack key enzymes, such as fumarate reductase and succinyl-CoA:acetate CoA transferase. However, the free energy released (ΔG^0^′ = −35 kJ/mol) in SAO indicates that there is no such reaction, which can be coupled to ATP synthesis (ΔG^0^′ = +60 to 70 kJ/mol) [82] directly, no matter which metabolic reactions contribute to SAO.

All SAOB are potentially able to activate acetate to acetyl phosphate via acetate kinase (Figure 4). In the case of *S. schinkii* and *Th. phaeum*, phosphotransacetylase activity enables the acetyl moiety to enter the WL pathway (Figure 4), as strongly supported by transcriptomic data obtained for *S. schinkii* [28]. For *C. ultunense* Esp/BS^T^ and *P. lettingae*, the metabolic fate of the acetyl phosphate still needs to be explained. However, mRNA expression and enzyme activity studies clearly indicate the involvement of formyltetrahydrofolate synthetase in the acetate oxidation pathway of *S. schinkii*, *T. phaeum*, *C. ultunense* Esp/BS^T^, and *T. acetatoxydans* [27,28,29]. For *P. lettingae*, no such expression or activity data have been published so far.

### 4.3. Energy Conservation Associated with Syntrophic Acetate Oxidation

**Figure 6:**
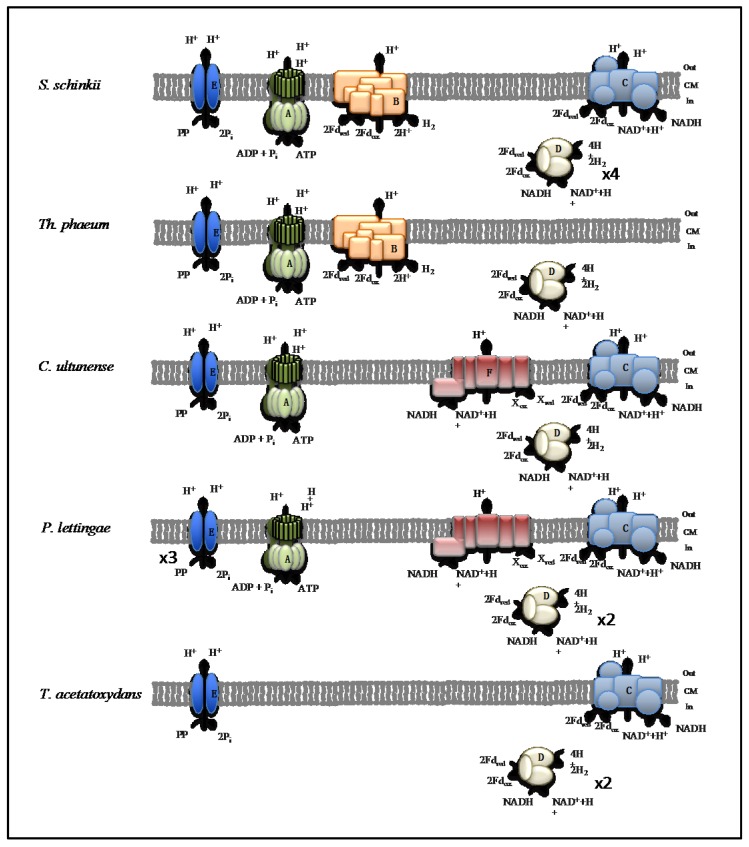
Energy conservation systems potentially involved in syntrophic acetate oxidation. (A) F_0_/F_1_ ATP synthase, (B) Ech (energy-converting hydrogenase) hydrogenase, (C) Proton-translocating ferredoxin:NAD^+^ oxidoreductase (Rnf) complex, (D) [Fe-Fe] hydrogenases, (E) ion-translocating pyrophosphatase, (F) NADH:quinone hydrogenase (NQR) complex. Numbers indicate predicted gene duplications. Na^+^ translocation might occur instead of H^+^ translocation.

The SAOB differ markedly in their energy conserving systems, indicating different strategies. No core set could be identified, except for [Fe-Fe] hydrogenases, which are known to be involved in H_2_ evolution, and which are encoded by all SAOB genomes (Figure 6).

As observed for many anaerobic bacteria, ferredoxin and rubredoxin seem to be the main electron carriers in energy metabolism. Ferredoxin contains iron-sulphur clusters as an electron-mediating cofactor. Flavodoxins contain a tightly bound FMN as the electron-receiving component. The redox potential is strongly affected by the apoprotein [83]. Rubredoxin contains redox-active iron, but it lacks inorganic sulphide. In the case of *C. ultunense*, *Th. phaeum* and *P. lettingae*, flavodoxins can potentially substitute for ferredoxin, e.g., under iron shortage. Both ferredoxin and flavodoxin can serve as electron donors in reactions with standard redox potential as low as −500 mV, and can therefore serve as either an electron donor or an electron acceptor in hydrogen production or consumption [84]. The respiratory Ech hydrogenase and Rnf complex can both couple ferredoxin oxidation to proton translocation. Ech hydrogenase transfers electrons from ferredoxin to protons, and Rnf complex mediates electron transfer from ferredoxin to NAD^+^, covering the redox span from E^0^′ = −500 to −413 mV and E^0^′ = −500 to −320 mV, respectively [63,85]. The proton gradient that was established by either complex can be used for ATP production and for providing energy for endergonic reactions, such as oxidation of methyl THF to methylene THF by methylene THF reductase, as needed in the oxidative WL pathway. The Rnf complex is the most widespread electron transport complex in anaerobic microorganisms [63]. However, it appears unlikely that the complex supports energy conservation in the oxidative WL pathway, as the standard potential of the NADH that is produced might not be sufficiently negative to reduce protons. This is in agreement with the observed down regulation of the Rnf genes in *S. schinkii* when oxidising acetate and the lack of Rnf genes in the genome of *Th. phaeum* [28,32] (Figure 6).

Instead, energy conservation by the oxidative WL pathway most likely involves Ech hydrogenase, which can couple oxidation of ferredoxin directly to the formation of a proton gradient (Figure 6). However, the oxidative WL pathway produces NADH as redox equivalent from three enzymatic reactions (reaction 6, 7, 10; Figure 5), and only one reaction reduces ferredoxin (reaction 4, Figure 5) [86]. The genomes of *S. schinkii* and *Th. phaeum* are predicted to encode potential electron-bifurcating ferredoxin- and NAD^+^-dependent [Fe-Fe] hydrogenases [28,32], which might couple the endergonic formation of hydrogen from NADH to the exergonic formation of hydrogen from reduced ferredoxin (Figure 6). In that case, a proton motive force would be generated due to the cytosolic consumption of protons. Transcriptomic studies that were performed on *S. schinkii* support the involvement of Ech hydrogenase and bifurcating [Fe-Fe] hydrogenase in acetate oxidation [28]. For *Th. phaeum*, a potential link between formate oxidation and bifurcating hydrogen formation has also been suggested [32]. A similar gene organisation has been found in *C. ultunense* Esp/BS^T^, indicating the potential connection of both activities. With regard to *T. acetatoxydans*, SAO-related energy conservation could potentially be achieved by a bifurcating [Fe-Fe] hydrogenase [30], rather than by respiratory complexes, as no such complexes have been predicted, except for a putative Rnf complex (Figure 6). This might also apply for *C. ultunense* Esp/BS^T^ and *P. lettingae*: The proton motive force is very likely formed by chemical or scalar protons rather than vectoral protons, as next to Rnf complex both genomes harbour only a putative bifurcating [Fe-Fe] hydrogenase (Figure 6). However, both of the genomes encode a putative NQR complex, which indicates that another electron acceptor rather than protons can potentially be utilised. This respiratory complex transfers electrons from NADH to quinone covering a redox span between −320 and +90 mV. The net redox reaction is similar to that carried out by H^+^ pumping NADH-quinone oxidoreductase complex that is found in mitochondria and many respiring bacteria, but the complexes are not homologous [60].

In *Vibrio cholera*, the NQR complex generates first an electrochemical Na gradient, followed by formation of a proton motive force by a respiratory complex that is connected via quinol. Interestingly, in *C. ultunense* Esp/BS^T^ and in *P. lettingae*, the genome lacks the quinone-binding subunit NQRA, indicating that the final electron acceptor could potentially have a more negative redox potential than quinone. The last redox carrier is very likely FMN, the redox potential of which strongly depends on the apoprotein [83]. The NQR complex might follow downstream of the Rnf complex, both together forming a potential respiratory chain (Figure 6). However, the involvement of the NQR complex in acetate oxidation and the final inorganic or organic electron acceptor(s) still need to be uncovered. Interestingly, the genome of *A. metalliredigens*, the current closest sequenced relative of *C. ultunense* Esp/BS^T^, also encodes a putative NQR complex (Figure 3). *A. metalliredigens* has been described as a strictly anaerobic metal-reducing bacterium, only distantly related to other commonly studied iron-reducing microorganisms [87]. It can utilise lactate or acetate (in the presence of yeast extract) when any of the electron acceptors Fe(III)-citrate, Fe(III)-EDTA, Co(III)-EDTA, or Cr(VI) is present [87]. For *C. ultunense* Esp/BS^T^, acetate oxidation using Fe(III) as an electron acceptor has been excluded [24,25]. In *P. lettingae*, acetate oxidation has been observed in the presence of thiosulphate [22]. In order to provide reduced ferredoxin for anabolic and catabolic pathways, both the Rnf complex and Ech hydrogenases can reverse the electron flow using the electrochemical gradient and drive the thermodynamically unfavourable reduction of ferredoxin with NADH or hydrogen. In *T. acetatoxydans*, *C. ultunense* Esp/BS^T^, and *P. lettingae*, the Rnf complex might connect the NADH pool fed by reactions of acetate oxidation to hydrogen evolution by hydrogenases, which requires ferredoxin. The ETF complex seems not play a role in energy conservation connected to SAO. This complex is mainly involved in oxidation of specific substrate by means of dehydrogenase reactions [88]. In the the case of *C. ultunense* Esp/BS^T^ and *P. lettingae*, the activity is likely to be associated to the beta-oxidation of fatty acids, as it is encoded together with an acyl-dehydrogenase subunit. The ETF found encoded in the genome of *T. acetatoxydans* likely receives electrons from oxidation of lactate, as it is part of an operon encoding a lactate uptake permease and a lactate dehydrogenase subunit.

Moreover, additional energy might be conserved by the ion-translocating pyrophosphatase found in all SAOB (Figure 6). These PPases are integral membrane proteins that couple the hydrolysis of pyrophosphate (PP_i_) to the transport of monovalent cations (Na^+^/H^+^) against the electrochemical potential gradient, generating an ion motive force [89]. Thus, PPi that is formed as a by-product of biosynthetic reactions in which nucleoside triphosphates are converted to nucleoside monophosphates could be hydrolysed by the membrane-bound PPase and contribute to the formation of a proton motive force and subsequently to the synthesis of ATP. In *S. schinkii*, PPase expression was observed when oxidising acetate syntrophically [28].

## 5. Summary

Very little is known about the metabolic capacity and flexibility of SAOB, mainly being due to strict cultivation requirements and difficulties in reconstituting the thermodynamically unfavourable acetate oxidation process under laboratory conditions. Therefore, genome-based studies and comparisons are important, as they provide insights into questions regarding metabolic and physiological potential and restrictions, and they provide the basis for metabolic engineering, control, and monitoring. The results of the present study clearly indicate unexpected metabolic diversity between the known SAOB with regard to acetate oxidation, energy conservation, and metabolic flexibility, and a certain environmental robustness that is beneficial for AD processes. The metabolic diversity also implies differences in regulation of the SAO pathways with respect to potential inducers, repressors, and regulators, which has potential consequences regarding the abundance and activity of SAOB in anaerobic digestion processes.

## Figures and Tables

**Table 1:** Summary of synteny analysis (%) and comparison of inferred proteins (number of genes) of *C. ultunense* strain Esp to *C. ultunense* strain BS^T^, Alkaliphilus *metalliredigens*, acetogens (*Moorella thermoacetica*, *Acetobacterium woodii*) and syntrophic acetate-oxidising bacteria (SAOB) (*S. schinkii*, *T. acetatoxydans*, *Th. phaeum*, *P. lettingae*).

Organism	% Age	No. of Genes
*T. acetatoxydans* Re1	33.36	1128
*C. ultunense* BS^T^	98.00	3323
*S. schinkii* Sp3	27.68	936
*T. phaeum* DSM 12270	27.24	921
*T. lettingae* TMO	16.98	574
*A. metalliredigens* QYMF	43.39	1467
*M. thermoacetica* ATCC 39073	26.44	894
*A. woodii* DSM 1030	26.32	890

**Table 2:** Comparison of COG (Clusters of Orthologous Groups) analysis for the SAOB *C. ultunense* strain Esp, *C. ultunense* strain BS^T^, *T. acetatoxydans*, *Th. phaeum*, *P. lettingae*, and *S. schinkii*.

Organism	Total COGs (% Age)	Total No. Genes	Amino Acid Transport & Metabolism	Carbohydrate Transport & Metabolism	Energy Production & Conservation	Inorganic Ion Transport & Metabolism
*T. acetatoxydans* Re1	81.25	2158	10.80	9.11	5.91	3.87
*C. ultunense* strain Esp	79.41	2623	9.23	5.26	5.54	4.60
*C. ultunense* strain BS^T^	77.78	2594	9.05	5.18	5.54	4.58
*T. phaeum* PB	73.50	2263	9.09	3.24	6.78	4.48
*P. lettingae* TMO	85.39	1853	10.78	13.17	6.35	6.08
*S. schinkii* Sp3	75.07	2586	9.84	4.00	5.92	5.97

**Table 3:** Energy-conserving systems and related proteins predicted for the SAOB *C. ultunense* strain Esp, *C. ultunense* strain BS^T^, *T. acetatoxydans* strain Re1, *Th. Phaeum* strain PB, *P. lettingae* strain TMO, and *S. schinki* strain Sp3 [28,30,32].

	*T. acetatoxydans* Re1 (Mesophilic)	*S. schinkii* Sp3 (Mesophilic)	*C. ultunense* Strain Esp (BS^T^) (Mesophilic)	*T. phaeum* PB (Thermophilic)	*P. lettingae* TMO (Thermophilic)
Rnf complex (*rnf*CDGEAB)	TepiRe1_2026-_2031	SSCH_420047-420053	CUESP_2612-2617 (AZSU01_50145-50150)	-	Tlet_0286-_0291
NQR complex (*nqr*BCDEF)	-	-	CUESP_0375-0379 (AZSU01_10374-10378)	-	Tlet_0865-0869
NADH Ferredoxin-depending [Fe-Fe] hydrogenase	TepiRe1_2033-2037 TepiRe1_2699-2701	SSCH_90017-19 SSCH_210008-10 SSCH_60009-11 SSCH_1120014-15	CUESP_2849-2850 (AZSU_01_60160-60162)	Tph_18440-_18460	Tlet_0952-0957 Tlet_1518-1522
Ech hydrogenase (*ech*ABCDEF)	-	SSCH_170021-26	-	Tph_21310-_21360	-
Periplasmic [Ni-Fe] hydrogenase (Maturation proteins)	-	SSCH_30031-_30033 (SSCH_60028-_60030)	-	Tph_06350-_06370 (Tph_09180-_09210)	-
Cytoplasmic [Ni-Fe] hydrogenase	-	SSCH_370001-6	-	Tph_26880-_26930	-
Ion-translocating Ferredoxin-NADH oxidoreductase/heterodisulfide reductase complex	-	SSCH_160001-8	-	-	-
Cytoplasmic formate dehydrogenase	-	SSCH_1520002-3	CUESP_2852-2856 (AZSU01_60163-60167)	Tph_27280-_27290 Tph_18420-_18430	-
Membrane bound formate dehydrogenase	-	SSCH_1490003-6	-	Tph_15370-_15400	-
Formate hydrogen lyase	-	-	-	Tph_26250-26370	-
Electron transfer flavoprotein (*Etf*AB)	TepiRe1_2532-2533	-	CUESP_1366-1368 (AZSU01_20759-20761)	-	Tlet_1692-1694 Tlet_1832-1833
ATP synthase	-	SSCH_240003-240010	CUESP_1228-1235 (AZSU_20626-20633)	Tph_27340-27360	Tlet_0160-0167
Membrane bound Na/H-PPase	TepiRe1_2120	SSCH_1440001	CUESP_0504 (AZSU01_10499)	Tph_08020	Tlet_1947 Tlet_1318 Tlet_2017
Ferredoxins	TepRe1_0333 TepRe1_0615 TepRe1_1978	SSCH_100042 SSCH_450007 SSCH_530010 SSCH_760007 SSCH_1120013	CUESP_0098 CUESP_0771 CUESP_1024 CUESP_1759 CUESP_2851 (AZSU01_10096 AZSU01_20179 AZSU01_30304 AZSU01_60162)	Tph_08140 Tph_08570 Tph_09550 Tph_11190 Tph_15740 Tph_17670 Tph_18150 Tph_24780	Tlet_0408 Tlet_0921 Tlet_0956 Tlet_1345 Tlet_1467 Tlet_1761 Tlet_2059
Rubredoxin	TepRe1_0396	SSCH_180038	CUESP_0131 (AZSU01_10127)	Thp_07990 Thp_23390 Thp_23400	Tlet_1612
Flavodoxin	-	-	CUESP_0726 CUESP_1387 CUESP_2600 CUESP_0610 (AZSU01_20134 AZSU01_20779 AZSU01_50133)	Tph_01330 Thp_21710	Tlet_0030 Tlet_1248 Tlet_1568 Tlet_1577
Cytochrome	-	-	-	-	Tlet_1388

## Supplementary Materials

The following are available online at http://www.mdpi.com/2073-4425/9/4/225/s1.
