# Supplementary material for: Genome-Guided Analysis of Clostridium ultunense and Comparative Genomics Reveal Different Strategies for Acetate Oxidation and Energy Conservation in Syntrophic Acetate-Oxidising Bacteria
Source: Genes (Basel). 2018 Apr 23;9(4):225. doi: 10.3390/genes9040225 (PMC5924567; doi:10.3390/genes9040225)
Supplement: Supplementary file 1 [file genes-09-00225-s001.pdf]

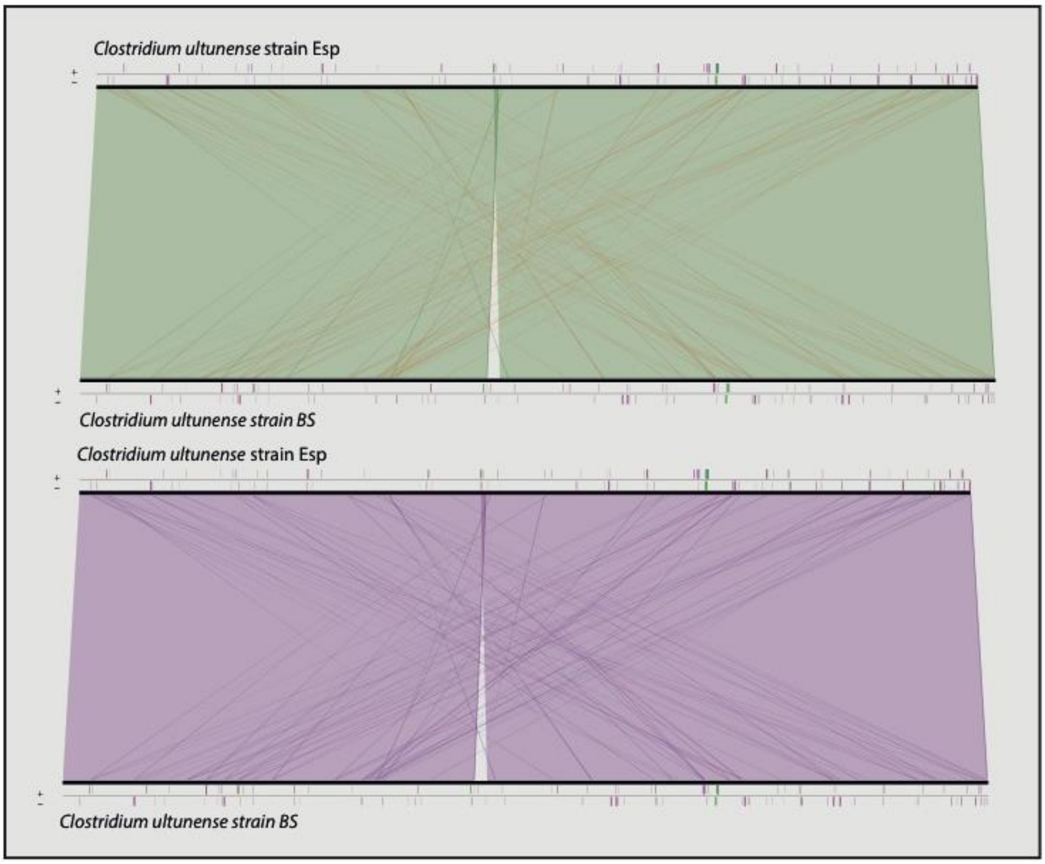

Figure S1. Synteny comparison of *Clostridium ultunense* strain Esp genome with the genome of the type strain *Clostridium ultunense* strain *BS*^{T}. The lines indicate syntons between two genomes. Red lines show inversions around the origin of replication. Vertical bars on the border line indicate different elements in genomes, where pink = transposases or insertion sequences, blue = rRNA and green = tRNA.

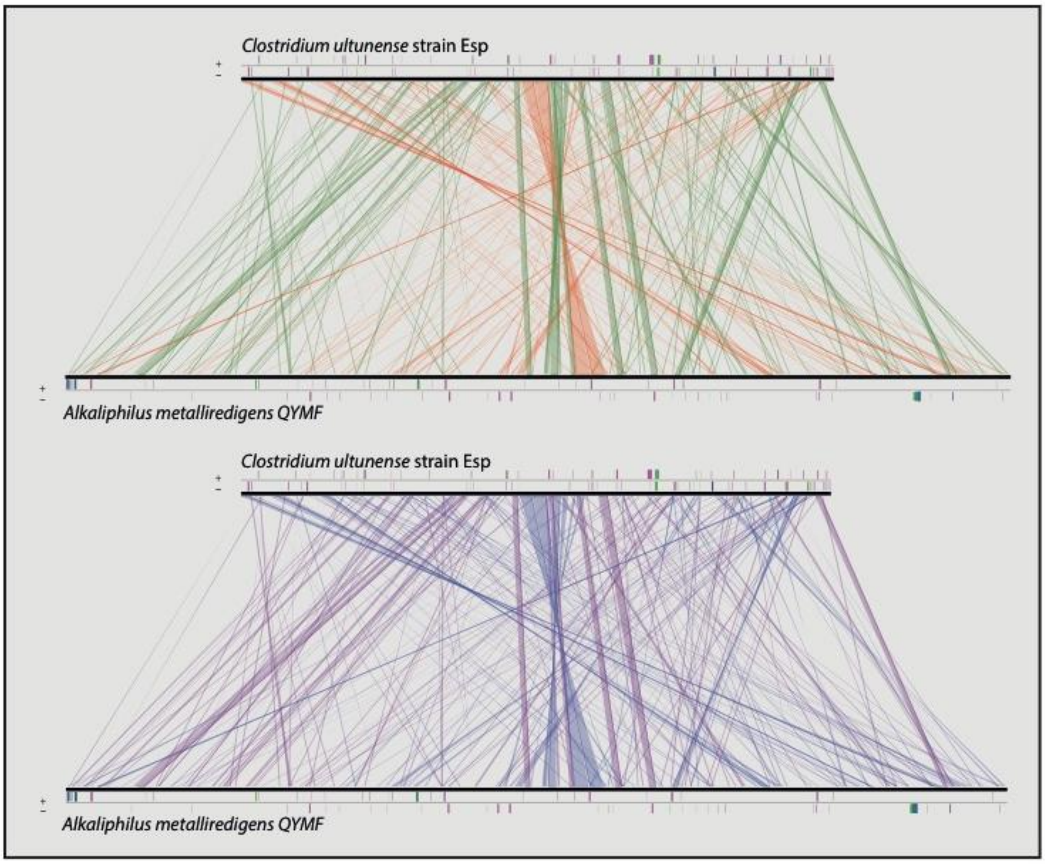

Figure S2. Synteny comparison of *Clostridium ultunense* strain Esp genome with the genome of *Alkaliphilus metalliredigens* QYMF. The lines indicate syntons between two genomes. Red lines show inversions around the origin of replication. Vertical bars on the border line indicate different elements in genomes, where pink = transposases or insertion sequences, blue = rRNA and green = tRNA.

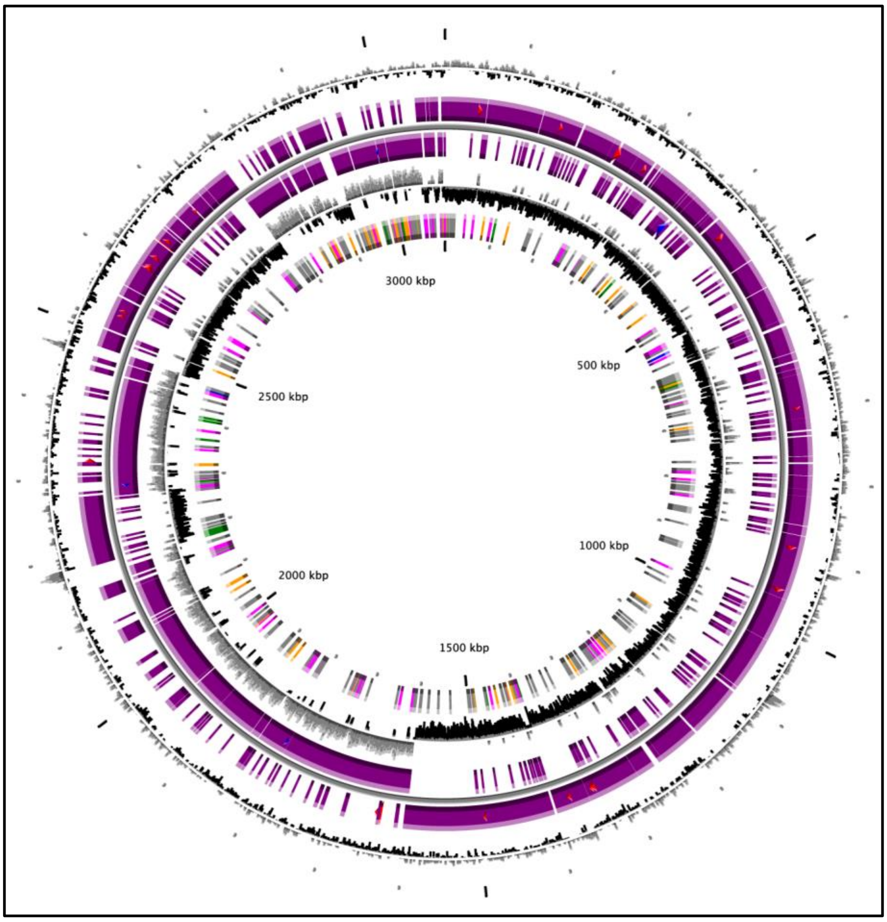

Figure S3. Circular map of the genome of *Clostridium ultunense* strain Esp. From outside to centre: (1) GC percent deviation (GC window - mean GC) in a 1000-bp window; (2) predicted CDSs transcribed in a clockwise direction; (3) predicted CDSs transcribed in a counter-clockwise direction; (4) GC skew (G+C/G-C) in a 1000-bp window; (5) rRNA (blue), tRNA (green), misc_RNA (orange), transposable elements (pink) and pseudogenes (grey). For more details see Table S1.

Table S1. Genome statistics for *Clostridium ultunense* Esp.

Attribute	Value	% of total
Genome size (bp)	3,093,245	100.00
DNA coding (bp)	2,629,258	85.00
DNA G+C content (bp)	9,89,838	32.40
Number of scaffolds	1	-
Total genes	3,303	100.00
Protein coding genes	3,236	97.97
RNA genes	66	1.25
Pseudo gene	57	1.99
Genes in internal clusters	1,580	45.00
Genes with function prediction	1,472	43.00
Genes assigned to COGs	2,623	79.41
Genes with Pfam domains	2,675	81.84
Genes with signal peptides	213	6.44
Genes with transmembrane helices	759	22.97
CRISPR repeats	8	0.24

Table S2. Presences of phages and phage remnants in *Clostridium ultunense* ESP, *Clostridium ultunense* BS^T, *Pseudothermotoga lettingae* TMO, *Syntrophaceticus schinkii* Sp3 and *Tepidanaerobacter acetatoxydans* Re1 genomes at time point of DNA isolation[1-3]. % of the genome which is predicted to encode prophage related genes are given in parenthesis.

**Clostridium ultunense ESP (3,33%)**						
Region	Region Length	Completeness	Total Proteins	Region position	Most Common Phage	GC%
1	7.7kb	Incomplete	9	86131-93902	PHAGE_Bacill_SP (NC_031245)	32.93%
2	24.4kb	Incomplete	21	1717007-1741436	PHAGE_Synech_ACG (NC_026927)	30.63%
3	29kb	Questionable	21	1813843-1842869	PHAGE_Mycoba_Panchino (NC_031281)	31.06%
4	6.8kb	Incomplete	9	2943158-2950029	PHAGE_Staphy_SPbeta (NC_029119)	32.17%
5	35.2kb	Intact	21	3051263-3086475	PHAGE_Stx2_c_1717 (NC_011357)	32.38%
**Clostridium ultunense BS^T (1,13%)**						
1	36.4kb	Intact	24	3172444-3208864	PHAGE_Stx2_c_1717 (NC_011357)	31.03%
**Pseudothermotoga lettingae TMO (1,7%)**						
1	11kb	Incomplete	14	999988-1010999	PHAGE_Plankt_LD (NC_016564)	37.88%
2	8kb	Incomplete	9	1115311-1123336	PHAGE_Synech_S_CAM7 (NC_031927)	39.22%
3	7.5kb	Incomplete	9	1465825-1473383	PHAGE_Geobac_E3 (NC_29073)	35.79%
4	10.3kb	Incomplete	10	1881355-1991711	PHAGE_Prochl_P_SSM2 (NC_006883)	39.65%
**Syntrophaceticus schinkii SP3 (1,8%)**						
1	4.7kb	Incomplete	7	88-4845	PHAGE_pSEUDO_JG004 (NC_019450)	48.97%
2	17.2kb	Incomplete	11	101385-118628	PHAGE_Bacill_Blue (NC_031056)	49.71%
3	8.1kb	Incomplete	9	2560898-2569090	PHAGE_Prochl_P_SSM2 (NC_006883)	51.39%
4	26.9kb	Incomplete	9	2846491-2873463	PHAGE_Plankt_PaV_LD (NC_016564)	43.36%
***Thermacetogenium phaeum* PB (1.45%)**						
1	42.8	Intact	41	2330541-2373372	PHAGE_Lactoc_28201 NC_031013	44.95%

1. Manzoor S, Bongcam-Rudloff E, Schnurer A, Muller B. Genome-Guided Analysis and Whole Transcriptome Profiling of the Mesophilic Syntrophic Acetate Oxidising Bacterium *Syntrophaceticus schinkii*. PLoS ONE. 2016;11(11):e0166520. Epub 2016/11/17. doi: 10.1371/journal.pone.0166520. PubMed PMID: 27851830; PubMed Central PMCID: PMCPMC5113046.
2. Müller B, Manzoor S, Niazi A, Bongcam-Rudloff E, Schnürer A. Genome-guided analysis of physiological capacities of *Tepidanaerobacter acetatoxydans* provides insights into environmental adaptations and syntrophic acetate oxidation. PLoS ONE. 2015;10(3):e0121237. doi: 10.1371/journal.pone.0121237.
3. Oehler D, Poehlein A, Leimbach A, Müller N, Daniel R, Gottschalk G, et al. Genome-guided analysis of physiological and morphological traits of the fermentative acetate oxidizer *Thermacetogenium phaeum*. BMC Genomics. 2012;13(1):723. doi: 10.1186/1471-2164-13-723.

Table S3. ABC transporter related genes found in *Clostridium ultunense* strain ESP.

Clostridium ultunense	**Product**
CUESP_0051	Energizing coupling factor of ABC influx transporter (ATP-binding protein)
CUESP_0052	Energizing coupling factor of ABC influx transporter (ATP-binding protein)
CUESP_0069	ABC transporter, substrate-binding lipoprotein
CUESP_0070	ABC transporter, permease protein
CUESP_0071	Nitrate ABC transporter
CUESP_0120	Oligopeptide ABC transporter, periplasmic oligopeptide-binding protein OppA (TC 3.A.1.5.1)
CUESP_0121	Oligopeptide ABC transporter (permease)
CUESP_0122	Oligopeptide ABC transporter (permease)
CUESP_0123	Oligopeptide ABC transporter (ATP-binding protein)
CUESP_0124	Oligopeptide ABC transporter (ATP-binding protein)
CUESP_0138	ABC transporter related protein
CUESP_0154	ABC transporter related
CUESP_0204	Cell-division ABC transporter (ATP-binding protein)
CUESP_0281	Bacitracin ABC efflux transporter (ATP-binding protein)
CUESP_0282	Efflux ABC transporter, permease protein
CUESP_0283	Putative ABC efflux transporter (ATP-binding protein)
CUESP_0284	Putative ABC-2 type transport system permease protein
CUESP_0303	Putative peptide ABC transporter permease protein y4tP
CUESP_0304	Putative peptide transporter permease subunit: membrane component of ABC superfamily
CUESP_0305	Oligopeptide ABC transporter (ATP-binding protein)
CUESP_0306	Oligopeptide ABC transporter (ATP-binding protein)
CUESP_0322	Phosphate ABC transporter ATP-binding protein
CUESP_0323	ABC transporter protein
CUESP_0365	Ribose ABC transporter (ATP-binding protein)
CUESP_0366	D-ribose transporter subunit ; membrane component of ABC superfamily
CUESP_0399	ABC transporter related
CUESP_0404	Putative ABC transporter permease protein
CUESP_0405	Putative ABC transporter permease protein
CUESP_0406	ABC transporter, ATP-binding protein
CUESP_0437	Putative permease of ABC transporter
CUESP_0438	Putative ABC transporter component, ATP-binding
CUESP_0443	ABC transporter related protein (fragment)
CUESP_0468	Sulfur mobilizing ABC protein, ATPase
CUESP_0493	Guanosine ABC transporter (ATP-binding protein)
CUESP_0495	Permease of ABC guanosine transporter
CUESP_0635	Phosphate ABC transporter (ATP-binding protein)
CUESP_0636	ABC transporter membrane-spanning permease-phosphate transport
CUESP_0637	Putative ABC transporter permease protein
CUESP_0686	Fragment of guanosine ABC transporter (ATP-binding protein) (part 1)
CUESP_0687	Fragment of guanosine ABC transporter (ATP-binding protein) (part 2)
CUESP_0731	Putative iron-siderophore ABC transporter (ATP-binding protein) (fragment)
CUESP_0816	Putative high-affinity branched-chain amino acid ABC transporter, permease protein LivH
CUESP_0817	Branched-chain amino acid ABC transporter, permease protein
CUESP_0818	Leucine/isoleucine/valine transporter subunit ; ATP-binding component of ABC superfamily
CUESP_0819	Leucine/isoleucine/valine transporter subunit ; ATP-binding component of ABC superfamily
CUESP_0922	Antibiotic ABC superfamily ATP binding cassette transporter

CUESP_0928	ABC transporter related
CUESP_0935	Guanosine ABC transporter (ATP-binding protein) (fragment)
CUESP_0936	Ribose ABC transporter (ATP-binding protein) (fragment)
CUESP_0941	Ribose ABC transporter (ATP-binding protein)
CUESP_0942	Fragment of ribose ABC transporter (permease) (part 1)
CUESP_0943	Fragment of ribose ABC transporter (permease) (part 2)
CUESP_1009	Putative ABC transporter permease protein HI_1471
CUESP_1011	ABC-type transporter, periplasmic subunit
CUESP_1037	Leucine/isoleucine/valine transporter subunit ; membrane component of ABC superfamily
CUESP_1039	Leucine/isoleucine/valine transporter subunit ; ATP-binding component of ABC superfamily
CUESP_1040	Leucine/isoleucine/valine transporter subunit ; ATP-binding component of ABC superfamily
CUESP_1053	Ribose ABC transporter (ATP-binding protein)
CUESP_1054	D-ribose transporter subunit membrane component of ABC superfamily
CUESP_1055	ABC-type sugar transport system periplasmic component-like protein
CUESP_1091	Putative ABC-type transport system, periplasmic component/surface lipoprotein
CUESP_1092	Uncharacterized ABC transporter ATP-binding protein YufO
CUESP_1093	ABC-type uncharacterized transport system,permease component
CUESP_1094	Putative ABC-type transport system, permease component
CUESP_1109	Oligopeptide ABC transporter (ATP-binding protein)
CUESP_1110	Putative cell wall oligopeptide ABC transporter (ATP binding protein)
CUESP_1135	Oligopeptide/dipeptide ABC transporter
CUESP_1136	Oligopeptide/dipeptide ABC transporter,permease protein
CUESP_1137	Oligopeptide ABC transporter (ATP-binding protein)
CUESP_1138	Putative cell wall oligopeptide ABC transporter (ATP binding protein)
CUESP_1139	Oligopeptide/dipeptide ABC transporter
CUESP_1334	ABC transporter-like protein
CUESP_1381	Putative ABC transporter (ATP-binding protein)
CUESP_1382	Putative Uncharacterized ABC transporter permease YvcS
CUESP_1398	Aliphatic sulfonate ABC transporter (ATP-binding protein)
CUESP_1419	ABC-type transport system, permease component
CUESP_1420	ABC transporter permease
CUESP_1434	ABC transporter related (fragment)
CUESP_1499	High affinity arginine ABC transporter (permease)
CUESP_1500	High affinity arginine ABC transporter (ATP-binding protein)
CUESP_1977	ABC transporter, permease protein
CUESP_1978	ABC transporter, substrate-binding protein
CUESP_2110	Putative ABC-type cobalt transport system permease component CbiQ and related transporters-like protein
CUESP_2111	ABC transporter related
CUESP_2112	ABC transporter, ATP-binding protein
CUESP_2242	Cobalt ABC transporter, inner membrane subunit CbiQ
CUESP_2249	ABC transporter related
CUESP_2251	Putative Iron compound ABC transporter,periplasmic iron compound-binding protein
CUESP_2252	Abc-2 transporter, permease protein
CUESP_2254	Iron-dicitrate ABC transporter (permease)
CUESP_2260	ABC transporter related protein
CUESP_2289	Uncharacterized ABC transporter ATP-binding protein YwjA
CUESP_2391	Fructose-amino acid ABC transporter (ATP-binding subunit) (fragment)
CUESP_2393	Polyamine transporter subunit ; membrane component of ABC superfamily
CUESP_2394	Spermidine/putrescine ABC transporter, permease protein 2
CUESP_2447	ABC-type transporter, periplasmic subunit
CUESP_2448	ABC-type transporter, integral membrane subunit

CUESP_2449	ABC-type transporter, integral membrane subunit
CUESP_2450	Oligopeptide ABC transporter (ATP-binding protein)
CUESP_2451	Oligopeptide ABC transporter (ATP-binding protein)
CUESP_2521	Methionine ABC transporter (ATP-binding protein)
CUESP_2522	Methionine ABC transporter, permease component
CUESP_2523	Methionine ABC transporter, substrate binding lipoprotein
CUESP_2635	ABC transporter, ATP-binding protein
CUESP_2761	Efflux ABC transporter, permease protein
CUESP_2804	ABC transporter related protein
CUESP_2814	Uncharacterized ABC transporter ATP-binding protein YvcR
CUESP_2830	Putative ABC transporter (ATP-binding protein)
CUESP_2831	ABC transport system permease protein
CUESP_2836	Putative ABC transporter, ATP-binding protein SagG
CUESP_2838	Putative ABC-2 type transporter
CUESP_2857	Putative ABC transporter anion-binding protein HVO_1888
CUESP_2858	Permease component of tungstate ABC transporter
CUESP_2859	ATPase component of tungstate ABC transporter (fragment)
CUESP_2879	ABC transporter related protein
CUESP_2880	Putative ABC transporter (ATP-binding protein)
CUESP_2884	Putative ABC transporter (ATP-binding protein)
CUESP_2887	ABC transporter related
CUESP_2917	ABC transporter substrate binding protein
CUESP_2918	Amino acid or sugar ABC transport system,permease protein
CUESP_2919	ABC transporter, ATP-binding protein
CUESP_2944	ABC transporter, ATP-binding protein (fragment)
CUESP_2945	ABC transporter, ATP-binding protein (fragment)
CUESP_2970	ABC transporter, ATP-binding protein
CUESP_3106	Putative Antibiotic ABC transporter, permease protein
CUESP_3127	ABC-type multidrug transport system, ATPase component
CUESP_3228	Oligopeptide ABC transporter (ATP-binding protein)
CUESP_3229	Oligopeptide ABC transporter (ATP-binding protein)
CUESP_3230	Oligopeptide ABC transporter (permease)
CUESP_3231	Oligopeptide ABC transporter (permease)
CUESP_3232	ABC-type transporter, periplasmic subunit
CUESP_3256	Fragment of putative ABC transporter component (part 1)
CUESP_3259	Fragment of putative ABC transporter component (part 2)
CUESP_3335	Putative ABC transporter permease protein HI_1471
CUESP_3337	ABC-type transporter, periplasmic subunit

Table S4. ABC transporter related genes found in *Pseudothermotoga lettingae* TMO and *Tepidanaerobacter acetatoxydans* genomes.

Pseudothermotoga lettingae	**Product**
Tlet_0021	ABC transporter-like protein
Tlet_0022	Polar amino acid ABC transporter inner membrane subunit
Tlet_0023	Polar amino acid ABC transporter inner membrane subunit
Tlet_0075	Oligopeptide/dipeptide ABC transporter ATPase
Tlet_0076	Oligopeptide/dipeptide ABC transporter ATPase
Tlet_0086	ABC transporter-like protein
Tlet_0137	ABC transporter-like protein
Tlet_0184	ABC transporter-like protein
Tlet_0198	Oligopeptide/dipeptide ABC transporter ATPase
Tlet_0199	Oligopeptide/dipeptide ABC transporter ATPase
Tlet_0211	ABC transporter-like protein
Tlet_0212	ABC transporter-like protein
Tlet_0296	ABC transporter-like protein
Tlet_0337	ABC transporter-like protein
Tlet_0353	Oligopeptide/dipeptide ABC transporter ATPase
Tlet_0354	Oligopeptide/dipeptide ABC transporter ATPase
Tlet_0360	ABC transporter-like protein
Tlet_0391	ABC transporter-like protein
Tlet_0463	ABC transporter
Tlet_0483	Polar amino acid ABC transporter inner membrane subunit
Tlet_0484	ABC transporter-like protein
Tlet_0485	ABC transporter-like protein
Tlet_0550	ABC transporter-like protein
Tlet_0551	ABC transporter
Tlet_0552	ABC transporter
Tlet_0644	Oligopeptide/dipeptide ABC transporter ATPase
Tlet_0645	Oligopeptide/dipeptide ABC transporter ATPase
Tlet_0647	Oligopeptide/dipeptide ABC transporter ATPase
Tlet_0648	Oligopeptide/dipeptide ABC transporter ATPase
Tlet_0677	ABC transporter-like protein
Tlet_0721	ABC transporter-like protein
Tlet_0737	ABC transporter-like protein
Tlet_0802	ABC transporter-like protein
Tlet_0803	ABC transporter-like protein
Tlet_0808	ABC transporter-like protein
Tlet_0837	ABC transporter-like protein
Tlet_0839	Anion ABC transporter anion-binding protein
Tlet_0856	Oligopeptide/dipeptide ABC transporter ATPase
Tlet_0857	Oligopeptide/dipeptide ABC transporter ATPase
Tlet_0861	Polar amino acid ABC transporter inner membrane subunit
Tlet_0862	ABC transporter-like protein
Tlet_0863	Polar amino acid ABC transporter inner membrane subunit
Tlet_0982	ABC transporter-like protein
Tlet_1020	ABC transporter-like protein
Tlet_1041	ABC transporter
Tlet_1042	ABC transporter-like protein
Tlet_1045	ABC transporter-like protein
Tlet_1062	ABC transporter-like protein
Tlet_1071	ABC transporter-like protein
Tlet_1072	ABC transporter-like protein
Tlet_1122	ABC transporter-like protein
Tlet_1123	ABC transporter-like protein
Tlet_1210	ABC transporter-like protein

Tlet_1211	ABC transporter-like protein
Tlet_1212	ABC transporter-like protein
Tlet_1217	ABC transporter-like protein
Tlet_1218	ABC transporter-like protein
Tlet_1234	Multidrug ABC transporter ATPase-like protein
Tlet_1234	Multidrug ABC transporter ATPase-like protein
Tlet_1235	ABC transporter
Tlet_1249	ABC transporter-like protein
Tlet_1266	ABC transporter-like protein
Tlet_1273	ABC transporter-like protein
Tlet_1280	ABC transporter-like protein
Tlet_1299	ABC transporter substrate-binding protein
Tlet_1309	Oligopeptide/dipeptide ABC transporter ATPase
Tlet_1310	Oligopeptide/dipeptide ABC transporter ATPase
Tlet_1320	ABC transporter-like protein
Tlet_1327	ABC transporter-like protein
Tlet_1342	ABC transporter-like protein
Tlet_1353	ABC transporter-like protein
Tlet_1372	ABC transporter-like protein
Tlet_1415	ABC transporter-like protein
Tlet_1416	ABC transporter-like protein
Tlet_1419	ABC transporter-like protein
Tlet_1441	Oligopeptide/dipeptide ABC transporter ATPase
Tlet_1442	Oligopeptide/dipeptide ABC transporter ATPase
Tlet_1471	ABC transporter-like protein
Tlet_1503	Oligopeptide/dipeptide ABC transporter ATPase
Tlet_1504	Oligopeptide/dipeptide ABC transporter ATPase
Tlet_1527	Oligopeptide/dipeptide ABC transporter ATPase
Tlet_1528	Oligopeptide/dipeptide ABC transporter ATPase
Tlet_1560	ABC transporter-like protein
Tlet_1561	Putative ABC-2 type transport system permease
Tlet_1608	ABC transporter-like protein
Tlet_1609	ABC transporter-like protein
Tlet_1641	ABC transporter-like protein
Tlet_1678	ABC transporter-like protein
Tlet_1699	Phosphate ABC transporter ATPase
Tlet_1791	ABC transporter
Tlet_1792	ABC transporter-like protein
Tlet_1834	ABC transporter
Tlet_1835	ABC transporter-like protein
Tlet_1861	Putative ABC-2 type transport system permease
Tlet_1862	ABC transporter-like protein
Tlet_1933	Oligopeptide/dipeptide ABC transporter ATPase
Tlet_1934	Oligopeptide/dipeptide ABC transporter ATPase
Tlet_1937	ABC transporter-like protein
Tlet_1938	ABC transporter-like protein
Tlet_2004	Oligopeptide/dipeptide ABC transporter ATPase
Tlet_2005	Oligopeptide/dipeptide ABC transporter ATPase
Tlet_2022	Oligopeptide/dipeptide ABC transporter ATPase
Tlet_2023	Oligopeptide/dipeptide ABC transporter ATPase
Tepidanaerobacter acetatoxydans	
TepiRe1_0051	Gluconate transporter
TepiRe1_0060	para-aminobenzoyl-glutamate transporter
TepiRe1_0066	Citrate transporter
TepiRe1_0232	Energy-coupling factor transporter ATP-binding protein EcfA 2
TepiRe1_0235	component of the influx ECF transporters
TepiRe1_0316	Efflux transporter, RND family, MFP subunit

TepiRe1_0327	Na^+/melibiose symporter-like transporter
TepiRe1_0330	Sugar (Glycoside-Pentoside-Hexuronide) transporter
TepiRe1_0527	Proton-coupled thiamine transporter YuaJ
TepiRe1_0553	MgtC/SapB transporter
TepiRe1_0643	multiple sugar-binding transporter ATP-binding protein
TepiRe1_0760	Magnesium transporter
TepiRe1_0761	Magnesium transporter
TepiRe1_1041	Gluconate transporter
TepiRe1_1610	Efflux transporter, RND family, MFP subunit
TepiRe1_1673	Gluconate transporter
TepiRe1_1694	Na/Pi-cotransporter II-related protein
TepiRe1_1695	Na/Pi-cotransporter II-related protein
TepiRe1_1709	Tripartite ATP-independent periplasmic transporter DctQ component
TepiRe1_1744	Zinc transporter ZupT
TepiRe1_1755	Chromate transporter
TepiRe1_1756	Chromate transporter
TepiRe1_1775	Cation diffusion facilitator family transporter
TepiRe1_1787	Uncharacterized sodium-dependent transporter YocR
TepiRe1_1858	Efflux transporter, RND family, MFP subunit
TepiRe1_1930	Phosphonate-transporting ATPase
TepiRe1_1931	Efflux transporter, RND family, MFP subunit
TepiRe1_2032	putative formate transporter
TepiRe1_2108	Tripartite ATP-independent periplasmic transporter DctQ component
TepiRe1_2216	Phosphate transporter
TepiRe1_2303	Gluconate transporter
TepiRe1_2306	Gluconate transporter
TepiRe1_2464	Gluconate transporter
TepiRe1_2523	Uncharacterized sodium-dependent transporter YocR
TepiRe1_2685	Chromate transporter
TepiRe1_2686	Chromate transporter
TepiRe1_2689	Chromate transporter
TepiRe1_2690	Chromate transporter
TepiRe1_2758	Putative biotin transporter BioY

1. Manzoor S, Bongcam-Rudloff E, Schnurer A, Muller B. Genome-Guided Analysis and Whole Transcriptome Profiling of the Mesophilic Syntrophic Acetate Oxidising Bacterium Syntrophaceticus schinkii. PLoS ONE. 2016;11(11):e0166520. Epub 2016/11/17. doi: 10.1371/journal.pone.0166520. PubMed PMID: 27851830; PubMed Central PMCID: PMCPMC5113046.
2. Oehler D, Poehlein A, Leimbach A, Müller N, Daniel R, Gottschalk G, et al. Genome-guided analysis of physiological and morphological traits of the fermentative acetate oxidizer Thermacetogenium phaeum. BMC Genomics. 2012;13(1):723. doi: 10.1186/1471-2164-13-723.

Table S5. ABC transporter related genes found in *Thermacetogenium phaeum* and *Syntrophaceticus schinkii* genomes [1].

Thermacetogenium phaeum	**Product**
Tph_c00470	ABC transporter permease
Tph_c01060	ABC transporter substrate-binding protein
Tph_c01070	ABC transporter, permease protein, FecCD family
Tph_c01080	ABC transporter ATP-binding protein
Tph_c01190	ABC transporter permease
Tph_c01200	ABC transporter ATP-binding protein
Tph_c01210	ABC transporter substrate-binding protein
Tph_c01550	ABC transporter permease
Tph_c01560	ABC transporter ATP-binding protein
Tph_c01570	ABC transporter substrate-binding protein
Tph_c01600	Molybdate ABC transporter substrate-binding protein ModA
Tph_c03230	ABC transporter ATP-binding protein
Tph_c03240	Cobalt ABC transporter permease CbiQ
Tph_c03410	Iron ABC transporter substrate binding protein
Tph_c03420	ABC transporter, permease protein FecCD
Tph_c03430	Iron ABC transporter ATP-binding protein
Tph_c03500	ABC transporter
Tph_c03550	Oligopeptide ABC transporter ATP-binding protein
Tph_c03560	Oligopeptide ABC transporter ATP-binding protein
Tph_c03980	ABC-type tungstate transport system, permease component TupB
Tph_c04360	ABC transporter ATP-binding protein
Tph_c04370	ABC-type cobalt import system, permease component
Tph_c04860	Phosphate ABC transporter ATP-binding protein PstB
Tph_c04880	Phosphate ABC transporter permease
Tph_c04890	Phosphate ABC transporter permease
Tph_c05200	Cobalt ABC transporter permease
Tph_c05210	Cobalt ABC transporter ATP-binding protein CbiO
Tph_c09780	Cobalt ABC transporter permease CbiQ
Tph_c09790	Cobalt ABC transporter ATP-binding protein CbiO
Tph_c10680	ABC transporter ATP-binding protein
Tph_c10690	ABC transport system permease
Tph_c13160	ABC transporter ATP-binding protein
Tph_c13800	Taurine ABC transport system permease protein TauC
Tph_c13810	Taurine ABC transporter ATP-binding protein TauB
Tph_c13820	Bicarbonate ABC transporter substrate binding protein
Tph_c15200	ABC-1-like protein kinase
Tph_c15800	Hemin ABC transporter ATP-binding protein HmuV
Tph_c15810	Vitamin B12 ABC transport system permease protein BtuC
Tph_c15820	Vitamin B12 ABC transporter substrate-binding protein BtuF
Tph_c17070	D,D-dipeptide ABC transporter ATP-binding protein DppF
Tph_c17080	D,D-dipeptide ABC transporter ATP-binding protein DppD
Tph_c17090	D,D-dipeptide ABC transport system permease protein DppC
Tph_c17100	D,D-dipeptide ABC transport system permease protein DdpB
Tph_c17110	D,D-dipeptide ABC transporter substrate binding protein DdpA
Tph_c17920	Glutathione ABC transporter substrate protein GsiD
Tph_c17930	Glutathione ABC transport system permease protein GsiC
Tph_c17940	Glutathione ABC transport system permease protein GsiA
Tph_c17950	Glutathione ABC transporter ATP-binding protein GsiA
Tph_c18310	Cobalt import ABC transporter substrate-binding protein
Tph_c18320	Cobalt import ABC transporter, permease protein
Tph_c19110	ABC transporter-like protein
Tph_c19120	ABC transporter ATP-binding protein YfiB

Tph_c20380	ABC-type glycine betaine transport system, substrate-binding domain-containing protein
Tph_c20470	ABC transporter permease protein YknZ
Tph_2236	ABC transporter related protein (fragment)
Tph_c22400	ABC-3 family transport protein
Tph_c23550	ABC transporter
Tph_c23560	ABC transporter
Tph_c23600	Antimicrobial peptide ABC transporter ATPase
Tph_c23660	ABC transporter permease
Tph_c23750	Branched chain amino acid ABC transporter permease
Tph_c24810	ABC transporter ATP-binding protein
Tph_c24820	ABC transporter ATP-binding protein
Tph_c25200	Oligopeptide/dipeptide ABC transporter ATPase
Tph_c25210	ABC-2-like transporter, permease protein
Tph_c26400	Molybdate/tungstate ABC transporter substrate binding protein ModA
Tph_c26410	Molybdate/tungstate ABC transport system permease protein ModB
Tph_c26420	Molybdate/tungstate ABC transporter ATP-binding protein ModC
Tph_c27340	Phosphate ABC transporter permease
Tph_c28160	Serine protease/ABC transporter B family protein TagC
Tph_c29150	ABC transporter ATP-binding protein
Syntrophaceticus schinkii	
SSCH_30006	ABC transporter polyketide tetronasin permease (fragment)
SSCH_60007	Component of SufBCD complex, ATP-binding component of ABC superfamily
SSCH_80005	Phosphate ABC transporter, inner membrane subunit PstA
SSCH_80006	Phosphate ABC transporter, inner membrane subunit PstC
SSCH_80011	Phosphate ABC transporter (ATP-binding protein)
SSCH_110002	Leucine/isoleucine/valine transporter subunit ; membrane component of ABC superfamily
SSCH_120026	Excinuclease ABC (subunit C) (part 2)
SSCH_120027	Exonuclease ABC subunit C (Part 1)
SSCH_120028	Excinuclease ABC (subunit A)
SSCH_120029	Fragment of excinuclease ABC (subunit B) (part 2)
SSCH_120030	Fragment of excinuclease ABC (subunit B) (part 1)
SSCH_150006	ABC transporter related
SSCH_160011	Putative iron transport protein (ABC superfamily, membrane)
SSCH_160012	ABC-type cobalamin/Fe3+-siderophore transport system, ATPase component
SSCH_160014	ABC-type Fe3+-hydroxamate transport system, periplasmic component
SSCH_160016	Putative iron transport protein (ABC superfamily, membrane)
SSCH_160017	Uncharacterized ABC transporter ATP-binding protein HI_1272
SSCH_190010	ABC transporter related protein
SSCH_200004	ABC-type transporter, periplasmic subunit (fragment)
SSCH_200005	Daunorubicin resistance ABC transporter ATPase subunit
SSCH_200008	ABC-type transporter, periplasmic subunit
SSCH_200010	ABC transporter related protein
SSCH_200011	Oligopeptide ABC transporter, ATP-binding protein (fragment)
SSCH_200012	Oligopeptide ABC transporter, ATP-binding protein OppD (fragment)
SSCH_220026	Uncharacterized ABC transporter ATP-binding protein YthP
SSCH_250007	ABC-2 type transporter (fragment)
SSCH_250013	ABC-type transporter, periplasmic subunit
SSCH_250014	ABC-type transporter, integral membrane subunit
SSCH_250022	ABC transporter related protein (fragment)
SSCH_260013	ABC transporter (ATP-binding protein); efflux of cationic peptides
SSCH_270005	ABC transporter, ATP-binding protein
SSCH_270006	D-ribose transporter subunit ; membrane component of ABC superfamily

SSCH_300015	Uncharacterized ABC transporter ATP-binding protein TM_0352 (fragment)
SSCH_340008	Leucine/isoleucine/valine transporter subunit ; ATP-binding component of ABC superfamily
SSCH_340009	ABC-type branched-chain amino acid transport systems, ATPase component
SSCH_340010	ABC-type branched-chain amino acid transport system, periplasmic component
SSCH_340011	ABC-type branched-chain amino acid transport system, permease component
SSCH_340012	Branched-chain amino acid ABC-type transport system, permease component
SSCH_350013	High affinity arginine ABC transporter (ATP-binding protein)
SSCH_350014	Glutamine transporter subunit ; membrane component of ABC superfamily
SSCH_350015	Glutamine transporter subunit ; periplasmic binding component of ABC superfamily
SSCH_380001	Fragment of putative dipeptide transporter (ABC superfamily, ATP_bind) (part 2)
SSCH_380002	Fragment of putative dipeptide transporter (ABC superfamily, ATP_bind) (part 1)
SSCH_380005	Putative peptide transporter permease subunit: membrane component of ABC superfamily
SSCH_380006	Nickel transporter subunit ; membrane component of ABC superfamily
SSCH_380008	ABC transporter related protein
SSCH_380026	DL-methionine transporter subunit ; periplasmic-binding component of ABC superfamily
SSCH_380027	Fragment of methionine ABC transporter, permease component (part 2)
SSCH_380028	Fragment of methionine ABC transporter, permease component (part 1)
SSCH_380029	Methionine ABC transporter (ATP-binding protein)
SSCH_390018	Putative ABC-type nitrate/sulfonate/bicarbonate transport systems, periplasmic components
SSCH_470002	Uncharacterized ABC transporter ATP-binding protein YufO
SSCH_470004	Nucleoside ABC transporter membrane protein (fragment)
SSCH_470005	Putative transport protein (ABC superfamily, membrane)
SSCH_500015	Putative ABC transporter ATP-binding protein BCE_3323 (fragment)
SSCH_520009	Phosphate ABC transporter membrane protein 2, PhoT family
SSCH_520010	Phosphate ABC transporter membrane protein 1, PhoT family (fragment)
SSCH_520064	Component of RuvABC resolvasome, endonuclease
SSCH_530001	Oligopeptide ABC transporter (permease) (fragment)
SSCH_540009	Oligopeptide/dipeptide ABC transporter, ATPase subunit
SSCH_540011	ABC-type transporter, integral membrane subunit
SSCH_540012	ABC-type transporter, integral membrane subunit
SSCH_540013	ABC-type transporter, periplasmic subunit (fragment)
SSCH_540014	ABC-type transporter, periplasmic subunit (fragment)
SSCH_560020	ABC-type proline/glycine betaine transport systems, permease component(fragment 1)
SSCH_560021	Putative transporter subunit: ATP-binding component of ABC superfamily transporter
SSCH_560023	Periplasmic glycine betaine/choline-binding (Lipo)protein of an ABC-type transport system (Osmoprotectant binding protein)
SSCH_600044	ABC-1 domain protein
SSCH_660007	ABC transporter, ATPase, predicted (fragment)
SSCH_660008	ABC transporter, ATPase (fragment)
SSCH_680012	Putative anion ABC transporter, solute-binding protein
SSCH_680013	Molybdate ABC transporter, permease protein
SSCH_680015	ABC-type transporter, periplasmic subunit (fragment)
SSCH_710008	ABC transporter related

SSCH_730003	ABC-type transporter, periplasmic subunit
SSCH_730007	ABC-2 type transporter
SSCH_730008	ABC-2 type transporter
SSCH_730023	Putative peptide transporter permease subunit: membrane component of ABC superfamily (Part 1)
SSCH_730024	Putative peptide transporter permease subunit: membrane component of ABC superfamily (Part 2)
SSCH_730025	Putative Dipeptide ABC transporter, dipeptide-binding protein
SSCH_730026	Oligopeptide/dipeptide ABC transporter, ATPase subunit
SSCH_730027	Putative peptide transporter permease subunit: membrane component of ABC superfamily
SSCH_750007	Fragment of oligopeptide ABC transporter (permease) (part 1)
SSCH_750008	Fragment of oligopeptide ABC transporter (permease) (part 2)
SSCH_750009	Dipeptide transporter ; membrane component of ABC superfamily
SSCH_750010	Putative peptide ABC transporter ATP-binding protein y4tR
SSCH_750011	Oligopeptide transporter subunit ; ATP-binding component of ABC superfamily
SSCH_760014	ABC transporter related
SSCH_760015	Uncharacterized ABC transporter permease protein YvrB
SSCH_770009	Oligopeptide ABC transporter (ATP-binding protein)
SSCH_770010	Oligopeptide ABC transporter (ATP-binding protein)
SSCH_770011	Dipeptide ABC transporter (permease)
SSCH_770012	Oligopeptide ABC transporter (permease) (fragment)
SSCH_790008	Cobalt ABC transporter, inner membrane subunit CbiQ
SSCH_810026	ABC transporter substrate binding protein
SSCH_810027	ABC-type transporter, integral membrane subunit
SSCH_810042	ABC transporter related protein
SSCH_850003	Putative ABC-type transport system, permease component
SSCH_870010	Cobalt ABC transporter, inner membrane subunit CbiQ (fragment)
SSCH_960033	Leucine/isoleucine/valine transporter subunit ; ATP-binding component of ABC superfamily
SSCH_960034	Leucine/isoleucine/valine transporter subunit ; ATP-binding component of ABC superfamily
SSCH_1060012	ABC-3 protein
SSCH_1060014	ABC-type metal ion transporter, periplasmic subunit
SSCH_1110003	ABC-type Fe3+ transport system periplasmic component-like protein
SSCH_1130011	ABC transport system permease protein
SSCH_1130012	ABC transporter substrate-binding protein
SSCH_1150029	ABC-type transporter, periplasmic subunit
SSCH_1210009	Putative multiple sugar ABC transporter (ATP-binding protein)
SSCH_1220015	Putative ABC-2 type transporter
SSCH_1220019	Putative ABC transporter related protein
SSCH_1290008	ABC transporter, ATP-binding protein
SSCH_1290009	ABC transporter, ATP-binding protein
SSCH_1290015	Uncharacterized ABC transporter ATP-binding protein YhaQ
SSCH_1290016	ABC-type Na^+ efflux pump permease component-like protein
SSCH_1350006	ABC-type cobalt transport system, ATPase component (fragment 2)
SSCH_1350007	ABC-type cobalt transport system, ATPase component (fragment 1)
SSCH_1350009	ABC transporter, permease and ATP-binding protein (fragment)
SSCH_1350019	Putative ABC-2 type transporter, permease protein
SSCH_1350024	Putative ABC-2 type transporter, permease protein
SSCH_1400002	Putative Cobalt ABC transporter, inner membrane subunit CbiQ
SSCH_1470005	ABC transporter related protein
SSCH_1520006	Molybdate transporter subunit ; membrane component of ABC superfamily
SSCH_1520007	Molybdate transporter subunit ; periplasmic-binding component of ABC superfamily

SSCH_1520012	ABC transporter related protein (fragment)
SSCH_1520013	ABC transporter related protein (fragment)
SSCH_1560001	Putative ABC transporter (ATP-binding protein) (fragment)
SSCH_1560002	Predicted ABC transporter, permease component (fragment)
SSCH_1560004	Predicted ABC transporter, permease component (fragment)
SSCH_1560005	Predicted ABC transporter, ATPase component
SSCH_1690004	Cell-division ABC transporter (ATP-binding protein)
SSCH_1730003	Uncharacterized ABC transporter ATP-binding protein YknY (fragment)
SSCH_1780002	Periplasmic glycine betaine/choline-binding (Lipo)protein of an ABC-type transport system (Osmoprotectant binding protein) (fragment)
SSCH_1870001	ABC-type multidrug transport system, ATPase component (fragment)
SSCH_1870003	ABC-2 type transporter (Part 1)
SSCH_1870004	ABC-2 type transporter (Part 2)
SSCH_1950003	Fragment of ABC transporter (ATP-binding protein) (part 2)
SSCH_1950004	Fragment of ABC transporter (ATP-binding protein) (part 1)
SSCH_2060002	ABC transporter family protein (fragment)
SSCH_2060004	Uncharacterized ABC transporter ATP-binding protein TM_0352 (fragment)
SSCH_2410001	ABC-type branched-chain amino acid transport system, periplasmic component (fragment)
SSCH_2620001	Uncharacterized ABC transporter ATP-binding protein TM_0352 (fragment)
SSCH_2930001	Uncharacterized ABC transporter ATP-binding protein YknY (fragment)

1. Manzoor S, Bongcam-Rudloff E, Schnurer A, Muller B. Genome-Guided Analysis and Whole Transcriptome Profiling of the Mesophilic Syntrophic Acetate Oxidising Bacterium Syntrophaceticus schinkii. PLoS ONE. 2016;11(11):e0166520. Epub 2016/11/17. doi: 10.1371/journal.pone.0166520. PubMed PMID: 27851830; PubMed Central PMCID: PMCPMC5113046.
